# Angiotensin-(1–7) ameliorates sepsis-induced cardiomyopathy by alleviating inflammatory response and mitochondrial damage through the NF-κB and MAPK pathways

**DOI:** 10.1186/s12967-022-03842-5

**Published:** 2023-01-02

**Authors:** Xin-Sen Chen, Jing-Rui Cui, Xiang-Long Meng, Shu-Hang Wang, Wei Wei, Yu-Lei Gao, Song-Tao Shou, Yan-Cun Liu, Yan-Fen Chai

**Affiliations:** Department of Emergency Medicine, Tianjin Medical University General Hospital, 154 Anshan Road, Tianjin, 300052 China

**Keywords:** Sepsis-induced cardiomyopathy, Mitochondria, Angiotensin (1–7), Angiotensin II, Oxidative stress, Apoptosis

## Abstract

**Background:**

There is no available viable treatment for Sepsis-Induced Cardiomyopathy (SIC), a common sepsis complication with a higher fatality risk. The septic patients showed an abnormal activation of the renin angiotensin (Ang) aldosterone system (RAAS). However, it is not known how the Ang II and Ang-(1–7) affect SIC.

**Methods:**

Peripheral plasma was collected from the Healthy Control (HC) and septic patients and Ang II and Ang-(1–7) protein concentrations were measured. The in vitro and in vivo models of SIC were developed using Lipopolysaccharide (LPS) to preliminarily explore the relationship between the SIC state, Ang II, and Ang-(1–7) levels, along with the protective function of exogenous Ang-(1–7) on SIC.

**Results:**

Peripheral plasma Ang II and the Ang II/Ang-(1–7) levels in SIC-affected patients were elevated compared to the levels in HC and non-SIC patients, however, the HC showed higher Ang-(1–7) levels. Furthermore, peripheral plasma Ang II, Ang II/Ang-(1–7), and Ang-(1–7) levels in SIC patients were significantly correlated with the degree of myocardial injury. Additionally, exogenous Ang-(1–7) can attenuate inflammatory response, reduce oxidative stress, maintain mitochondrial dynamics homeostasis, and alleviate mitochondrial structural and functional damage by inhibiting nuclear factor-kappa B (NF-κB) and mitogen-activated protein kinase (MAPK) signaling pathways, thus alleviating SIC.

**Conclusions:**

Plasma Ang-(1–7), Ang II, and Ang II/Ang-(1–7) levels were regarded as significant SIC biomarkers. In SIC, therapeutic targeting of RAAS, for example with Ang-(1–7), may exert protective roles against myocardial damage.

**Supplementary Information:**

The online version contains supplementary material available at 10.1186/s12967-022-03842-5.

## Introduction

Dysregulation of the host response by an established or suspected infection causes sepsis, which is a life-threatening organ dysfunction [1]. The heart is particularly susceptible to sepsis, and cardiac insufficiency elevates the likelihood of patient death [2]. Sepsis-induced cardiomyopathy (SIC) is an invertible myocardial depression disease mainly caused by myocardial energy metabolism disorder or direct myocardial injury [2, 3]. SIC mainly presents with systemic diastolic and/or systolic dysfunction [2, 3]. SIC occurs in about 44% of septic patients, and the relative risk of death more than doubles [4]. Although SIC is common in clinical practice, its pathogenesis has not been fully elucidated. Relevant studies suggest that the occurrence of SIC is related to oxidative stress, cardiomyocyte apoptosis, excessive inflammatory response, mitochondrial dysfunction, disturbance of calcium ion regulation, and autonomic dysfunction [5, 6]. Presently, there are no effective preventive and therapeutic measures for SIC [7]. Therefore, new therapeutic strategies are urgently needed.

Following the onset of sepsis, pathogenic microorganisms invade the body and trigger the intracellular signaling processes in immune cells, which allows them to synthesize and secrete several inflammatory factors that can cause myocardial damage [6]. Related studies have found that a large number of macrophages and neutrophils infiltrate the myocardial tissue during the early stages of SIC [8, 9]. However, nicotinamide adenine dinucleotide phosphate (NADPH) oxidase in macrophages and neutrophils can promote the intracellular production of reactive oxygen species (ROS), which can damage the cardiac myocytes and impair myocardial contractile function [10]. In addition, higher production of ROS promotes the translocation of nuclear factor-kappa B (NF-κB) from the cytoplasm to the nucleus, thereby increasing the release of pro-inflammatory cytokines [11]. ROS also disrupts the myocardial mitochondrial cell membrane, inhibits mitochondrial oxidative phosphorylation, and hinders the tricarboxylic acid cycle, thereby decreasing the adenosine triphosphate (ATP) production, which results in impaired energy supply to cardiac myocytes [12]. Intracellular apoptotic signaling pathways can be activated during the development of sepsis due to excessive inflammation, oxidative stress, and mitochondrial malfunctioning in cardiomyocytes, which further accelerate the apoptosis of cardiomyocytes [13]. As a result, SIC treatment strategies must focus on identifying solutions that can simultaneously decrease oxidative stress and inflammatory responses, and alleviate mitochondrial dysfunctioning.

The renin–angiotensin–aldosterone system (RAAS) balances the blood pressure and electrolyte concentration, and it also promotes the repair of damaged blood vessels [14]; regulates inflammatory responses, reduces oxidation stress, and maintains immune balance [15]. Therefore, RAAS is critical in sepsis-induced organ dysfunction. Being the core effector molecule of classical RAAS, Angiotensin (Ang) II is a powerful pro-inflammatory mediator [16]. Ang II Type 1 Receptor (AT1R), as a major Ang II receptor, can be found in the kidney, brain, heart, lung tissues, blood vessels, and immune cells [17]. In addition to inducing vasoconstriction, increased sympathetic activity, inflammatory cell infiltration, and fibrosis in associated organs, the Ang II/AT1R activation also increases the production of adhesion molecules and chemokine factors [18–22]. Angiotensin-Converting Enzyme 2 (ACE2) catalyzes the conversion of Ang II (a biologically active peptide in RAAS) to Ang-(1–7) [23, 24]. Mas Receptor (MasR) is a primary Ang-(1–7) receptor [25]. After binding to MasR, Ang-(1–7) blocks Ang II/AT1R function by exhibiting anti-apoptotic, vasodilatory, anti-inflammatory, anti-hypertrophic, and anti-fibrotic properties [25–28].

When sepsis occurs, the host Ang II/AT1R and the Ang-(1–7)/MasR axes become imbalanced, Ang II levels are elevated and it is associated with microvascular dysregulation and organ damage [29]. Additionally, Ang II can be regarded as the predictor of mortality in septic patients, and the earlier use of AT1R blockers (ARBs) could decrease short-term mortality in septic patients [30–34]. However, the clinical relevance and variations in the Ang-(1–7) and Ang II levels in septic patients with myocardial dysfunction complications have not been widely studied. In the past, a few researchers conducted animal experiments and noted that classical RAAS inhibitors can down-regulate the Ang II/AT1R axis to reduce sepsis-induced organ (such as kidney and lung) injuries [35–38]. Furthermore, additional animal experiments revealed that ACE2/Ang-(1–7)/MasR axis upregulation improves sepsis-related acute lung injury (ALI) by hindering the NF-κB and mitogen-activated protein kinase (MAPK) pathways [37, 39, 40]. The administration of exogenous Ang-(1–7) to mice lowers their Ang II levels, reduces inflammation, reduces oxidative stress, and alleviates sepsis-induced acute kidney injury (SIAKI) via the NF-κB pathway [41]. However, it is unclear whether the Ang-(1–7) or Ang II proteins are involved in SIC and whether the exogenous addition of Ang-(1–7) can alleviate SIC by inhibiting Ang II/AT1R axis.

This study's clinical component investigated the regularity of variations in Ang-(1–7) and Ang II concentrations among SIC patients and their relationship with myocardial injury in SIC patients. In addition, the animal and cell models of SIC were constructed by LPS to further explore whether Ang-(1–7) has a cardiac protective impact on SIC and its related molecular mechanisms. In conclusion, this research provides new theoretical support and an experimental base for designing new treatment strategies for SIC through a combination of clinical and basic research.

## Experimental procedures

### Chemicals

LPS (O111:B4, #L2630) was procured from Sigma-Aldrich Ltd. (St. Louis, USA). APExBIO (Houston, TX, USA) supplied Ang-(1–7) (#A1041; ≥ 99% purity) and A-779 (#B6056; ≥ 98% purity).

### Subjects

In this study, the sepsis (SIC and non-SIC) patients as well as the healthy control (HC) admitted to the General Hospital of Tianjin Medical University in the duration ranging from July 2021 to September 2022, were investigated. The septic patients satisfying all inclusion criteria were allowed to participate in this research: (1) Fulfilled the diagnostic criteria of Sepsis 3.0, and (2) Ages ranging from 18 to 80 years. In addition, the SIC patients who satisfied all inclusion criteria [7, 42] were involved in this research: (1) Satisfied the criteria for sepsis diagnosis; (2) Showed the Troponin I (TnI) concentrations > 0.4 ng/mL (wherein normal concentrations at the hospital ranged between 0 and 0.4 ng/mL) or brain natriuretic peptide (BNP) concentration > 100 pg/mL, within 24 h of admitting the patients, and (3) Showed the Left Ventricular Ejection Fraction (LVEF) less than 50% within 24 h of admitting the patients. On the other hand, the exclusion criteria for patients included: (1) Those suffering from a pre-diagnosed autoimmune disease, chronic heart failure, cardiomyopathy, liver failure, renal failure, or tumors; and (2) Aged less than 18 and above 80 years. Within 2 h of their admission, blood samples from all groups of patients were drawn, analyzed, and significant clinical data was noted. All members included in this research, or their families, provided informed consent, and the Ethics Committee members at the General Hospital of Tianjin Medical University were sent a copy of the experimental procedures performed in this study for approval.

### Animals and models

C57BL/6J male mice (weight 22–25 g) were procured from HFK Animal Science and Tech. Co. (China). All the animals were categorized randomly into 4 sets: (1) Control; (2) Ang-(1–7); (3) LPS; 4) LPS + Ang-(1–7). The Ang-(1–7) molecules were dissolved using saline. Based on the earlier studies, the intervention measures of Ang-(1–7) were 2 mg/kg Intraperitoneal (IP) injection every day for 3 consecutive days [43]. Referring to previous studies, a reproducible animal model of SIC was established by injecting LPS, (10 mg/kg; IP) [44, 45]. The Ang-(1–7) and control groups were IP-injected using Ang-(1–7) (2 mg/kg) and the same volume of solvent (saline) daily for 3 consecutive days. The animals in LPS and LPS + Ang-(1–7) groups were IP injected with saline or Ang-(1–7) every day for 3 consecutive days, and LPS (10 mg/kg) was IP injected, within 30 min, at the end of 3 days. The mouse survival rate was observed every 12 h after administering a lethal dose of LPS (20 mg/kg; IP) until 72 h. Additionally, echocardiography was performed 12 h after modeling. The Animal Ethics Committee in the General Hospital of Tianjin Medical University granted its approval to all animal research experiments, based on the National Institute of Health (NIH) regulations.

### Cell culture and treatments

The Chinese Academy of Sciences Cell Bank (China) provided the rat cardiomyocyte-derived cell line, i.e., H9c2, used in this study. DMEM medium with 1% (v/v) antibiotic solution and 10% (w/v) Fetal Bovine Serum (FBS) was used for the purpose of cultivating the H9c2 rat cardiomyocytes in the carbon dioxide incubator using 5% CO_2_ and 95% humidified air, at 37 °C. FBS (#SA211.02) was purchased at CellMax (Beijing, China). Using data published in the past, a SIC cell model was constructed using LPS (1 ug/mL) [45, 46]. To calculate the optimal intervention concentration, the H9c2 cells were initially pretreated using varying Ang-(1–7) concentrations (i.e., 10^−8^, 10^−7^, and 10^−6^ mol/L) for 1 h; and cells were either cultured with/without LPS for 12 h. Then, after identifying the optimum Ang-(1–7) intervention concentration, the H9c2 cells were classified into four distinct groups: LPS, LPS + Ang-(1–7), Ang-(1–7), and control.

### Cell viability

In a 96-well plate, H9c2 cells were inoculated (cell density of 5000 cells in every well), where the volume in every well was kept at 90 µL. When the H9c2 cells (in different groups) formed a monolayer at the bottom of the well, the corresponding drug treatment was given for the respective time durations. CCK-8 (10 µL; Dakewe, Beijing, China) was added to every well in the culture plate and the plates were further incubated in the incubator for 1.5 h. Lastly, the absorbance of every well was measured to calculate cell viability.

### Echocardiography

Firstly, the mice in every group were subjected to 1% isoflurane anesthesia and bound to the heating plate of the ultrasound instrument. The heating plate was set at 37 °C, and their chest hair was removed. Then, an ultrasound probe (Vevo2100; Fujifilm Visual Sonics, Canada) with a 30 MHz sampling frequency was used to capture the M-type motion curve from the mitral papillary muscle in mice and all corresponding indices were measured.

### Histologic analysis

The mice myocardial tissue specimens were fixed with 4% Paraformaldehyde (PFA), dehydrated using a sucrose gradient, embedded, and cut into 5 µm slices. Sections in each group were dewaxed using the alcohol gradient and stained using the Hematoxylin–Eosin stains (HE), and the histopathological differences in myocardial tissue were observed under the light microscope. The pathological damage score of myocardial tissues was based on the following parameters: myocardial tissue structure; myocardial fiber arrangement; whether the myocardial cell nucleus has obvious nuclear swelling and pyknosis; the presence of obvious inflammatory cell infiltration, edema, or congestion in the interstitial layer (all scored from 0 to 3). Both pathologists completed all histopathological scores in a double-blind manner.

### Immunofluorescence

Each myocardial tissue section was ruptured, blocked, and incubated at 4 °C, overnight using the F4/80 primary antibodies (Bioss, Beijing, China). Afterward, the thin slices were thoroughly rinsed using PBS for 10 min and incubated in the presence of Alexa Fluor 488-conjugated secondary antibodies (Beyotime, Shanghai, China) for 60 min. Lastly, the samples were analyzed with the aid of the fluorescence microscope (Olympus, Japan), and photomicrographs were acquired.

### Transmission electron microscopy (TEM)

The left ventricular myocardium of each group was cut into 1 mm^3^ pieces using a sterile blade and the pieces were fixed with 4% glutaraldehyde for 4 h and 1% osmium tetroxide for 2 h. Then, the fixed tissue was rinsed with PBS, dehydrated, embedded in paraffin, sectioned, and stained. The stained sections were micrographed at random locations using an H7500 TEM (Hitachi, Tokyo, Japan). Finally, the Flameng score method was used to semi-quantitatively analyze the mitochondrial damage in the cardiomyocytes from each group [47].

### Terminal deoxynucleotidyl transferase dUTP nick end labeling (TUNEL) assay

Myocardial specimens were stained using a commercial TUNEL kit (Elabscience, China). The myocardial tissue specimens were immersed in 4% PFA and fixed for 30 min, and washed twice with PBS, 5 min each. Proteinase K (100 µL) solution was pipetted to every sample and samples were incubated for 10 min at 37 °C, and rinsed with PBS twice. Then, the equilibrium buffer (100 µL) was added to every slice and the reaction was continued for 10 min. After removing the equilibration solution, the labeled working solution (50 µL) was added to each section, incubated for 60 min, in dark at 37 °C, and rinsed with PBS (3-times), and the tissue sections were sealed with the aid of the DAPI-containing reagents. The positive nucleus was seen to be green under the Olympus fluorescence microscope (Japan), and the no. of TUNEL positive cells/ unit area of myocardial tissue slices, in every group, was calculated.

### Dihydroethidium (DHE) staining

The DHE staining technique was employed to observe the concentration of reactive oxygen species (ROS) in H9c2 cells and myocardial tissues, based on the kit instructions (Beyotime, Shanghai, China). Briefly, a serum-free medium was used to prepare the working solution for the ROS probe at a concentration of 10 µm. Each myocardial tissue section was covered with this ROS probe working solution (30 µL) and incubated for 60 min, at 37 °C, in dark. Finally, the slices were photographed under the fluorescence microscope, and the Mean Fluorescence Intensity (MFI) was analyzed. The ROS detection method for H9c2 cells was as follows: The working solution of the ROS probe was used to resuspend H9c2 cells, and MFI was detected by flow cytometry after incubating them for 30 min, in dark, at 37 °C.

### Mitochondrial membrane potential (MMP) assay

A commercial JC-1 staining kit (Solarbio, Beijing, China) was employed to measure the MMP in myocardial tissue and H9c2 cells. The following MMP detection method was used for myocardial tissues: Initially, the mitochondrial organelles were extracted from the myocardial tissues by a kit (Solarbio, China), and the total protein concentration in the mitochondrial samples was estimated using the Bradford assay. Thereafter, the purified mitochondria (20 µg) were suspended in a dilute JC-1 working solution. Finally, the fluorescence intensity of the JC-1 aggregates and monomer was detected with a BioTek fluorescence microplate analyzer (Synergy HT; USA). Here, MMP can be defined as the ratio of red and green fluorescence intensities. The MMP in H9c2 cells is detected using the following method: The H9c2 cells from each group (after respective treatment) were collected, suspended in the JC-1 working solution, incubated for 20 min at 37 °C, and rinsed using the JC-1 buffer. Finally, they were analyzed using the flow cytometry technique.

### ATP content detection

A kit (Solarbio, Beijing, China) was used to detect the ATP concentration in the myocardial mitochondria, using the following steps: Initially, the myocardial tissue weights were recorded (approx. 0.1 g), and the tissues were suspended in the extract (1 mL), and homogenized. This homogenate was centrifuged at 800×*g* for 10 min at 4 °C, and Cell-Free Supernatant (CFS) was collected in a different tube. Chloroform (500 µL) was added to a tube, shaken thoroughly, and then centrifuged for 3 min, 10,000×*g*, at 4 °C. The clear supernatant was separated into another tube. Then, the OD of the supernatant was spectrophotometrically measured (Thermo Fisher Scientific, USA) at 340 nm before and after incubating the samples in a water bath at 37 °C. Finally, ATP content was estimated.

### Detection of cardiomyocyte apoptosis by flow cytometry

The AnnexinV-FITC kit (Beyotime, China) was employed for detecting the apoptosis of H9c2 cells. After treatment of H9c2 cells in each group, the cell suspension was buffer rinsed and centrifuged. The cell pellet was gently resuspended using the binding solution (195 µL) and Annexin V-FITC (3 µL) and propidium iodide (PI, 5 µL) stains were pipetted into the tubes, respectively, and mixed gently. Finally, the cells were allowed to incubate in dark, at room temperature (RT) for 20 min, and detected by means of the flow cytometry technique.

### Quantitative real-time PCR technique

Myocardial tissue (10 mg) was collected from every group and homogenized thoroughly. Then, the TRIzol reagent (TR201-50; Tianmo Biotech, China) was used for total RNA extraction. Thereafter, the concentration and purity of extracted RNA sample were determined and the reverse transcription commercial kit (KR118; Tiangen, Beijing, China) was employed for reverse transcribing the purified RNA into cDNA. This cDNA was used as a template, and the 2× SYBR Green Master Mix (B21203; Bimake, TX, USA) and the downstream and upstream primers of the target gene were sequentially added to the reaction mixture (20 µL), and amplified with the help of a CFX96 RT-PCR apparatus (Bio-Rad, USA). Primer sequences of internal reference and target genes are presented in Additional file 1: Table S1. Finally, the 2^−ΔΔCt^ method was employed for analyzing the quantitative data.

### Enzyme-linked immunosorbent assay (ELISA) procedure

The peripheral blood specimens, extracted from the clinical research subjects and mice, were centrifuged. The supernatant was collected and stored at − 80 °C till further use. Centrifuge tubes and cryopreservation tubes were purchased from NEST Biotechnology Co., Ltd. (Wuxi, China). The total protein content in myocardial tissue homogenates and H9c2 cells from every group were extracted and the total concentration was measured with a bicinchoninic acid (BCA) assay. The IL-1β, TNF-α, Ang-(1–7), IL-6, Ang II, and cTnT concentrations could be detected using the ELISA kit. IL-1β, TNF-α, and IL-6 ELISA kits were purchased from Dakewe (Beijing, China); while the Ang-(1–7), Ang II, and cTnT ELISA kits were purchased from Jingkang Bioengineering Co., Ltd (Shanghai, China).

### Western blotting

The myocardial tissue and H9c2 cells were collected, and RIPA lysate was added to homogenize them on ice with a homogenizer. After lysis, the supernatant was collected after centrifugation. Then, the concentration of total proteins in CFS was estimated by the BCA technique. First, total protein (40 µg) was pipetted in the SDS-PAGE apparatus and electrophoresed, transferred to the membrane, blocked, and incubated overnight using the corresponding primary antibody at 4 °C. This membrane was TBST-rinsed and incubated using the corresponding secondary antibodies at RT for 60 min. Eventually, the enhanced chemiluminescent solution (Millipore) was added drop-by-drop onto the protein strip for visualization using the imaging apparatus (Bio-Rad). All antibodies used for western blotting analysis were as follows: anti-β-actin (1:1000, #3700), anti-Bax (1:1000, #14796), anti-caspase-9 (1:1000, #9508), anti-Bcl-2 (1:1000, #3498), anti-P65 (1:1000, #8242), anti-cleaved caspase-3 (1:1000, #9664), and anti-p-P65 (1:1000, #3033) (purchased from Cell Signaling Technology, Danvers, USA); while anti-p-P38 (1:1000, #28,796–1-AP), anti-JNK (1:1000, #51151–1-AP), anti-P38 (1:1000, #14064–1-AP), anti-p-ERK (1:1000, #28733–1-AP), anti-p-JNK (1:1000, #80024-1-AP), and anti-ERK (1:1000, #16443–1-AP), were procured from Proteintech (Wuhan, China). Antibodies like anti-Drp1 (1:1000, #sc-271583), anti-Mfn2 (1:1000, #sc-515647), anti-IκBα (1:1000, #sc-1643), and anti-p-IκBα (1:1000, #sc-8404) were purchased from Santa Cruz Biotech Ltd (USA).

### Statistical analysis

The Shapiro–Wilk test was conducted for testing the normal data distribution. If the data fit into a normal distribution, it was expressed as an average value ± Standard Deviation (SD). Both groups were tested using a t-test. To compare numerous groups, a one-way ANOVA with the Bonferroni multiple comparison test was employed. If the data exhibited a non-normal distribution, the interquartile and median ranges were used. A Mann–Whitney test was utilized for the comparison of two groups, and comparisons across multiple groups were done with the Kruskal–Wallis and Dunn post-hoc multiple comparison procedures. The chi-squared test (χ^2^) was employed for comparing the groups for categorical variables, which are represented as frequency and percentage. Using Spearman's rank correlation coefficient, the correlation between the variables was expressed. The log-rank test was performed to assess the survival data, and the Kaplan–Meier (KM) curve was utilized to depict the cumulative survival rate. The Prism v8.0 statistical package (GraphPad, USA) was used for the above calculations. Values with P < 0.05 were considered significant.

## Results

### Clinical data comparison and correlation analysis of patients with sepsis

In total, 46 participants who satisfied the inclusion criteria were allowed to participate in this study, which included 34 septic (18 non-SIC and 16 SIC) and 12 HC patients. Additional file 2: Table S2 shows the clinical data of all the included subjects. Patients with sepsis exhibited lower platelet and lymphocyte counts and higher White Blood Cells (WBC), Total Bilirubin (TBIL), Aspartate aminotransferase (AST), creatinine and neutrophil levels in comparison to HC.

**Fig. 1:**
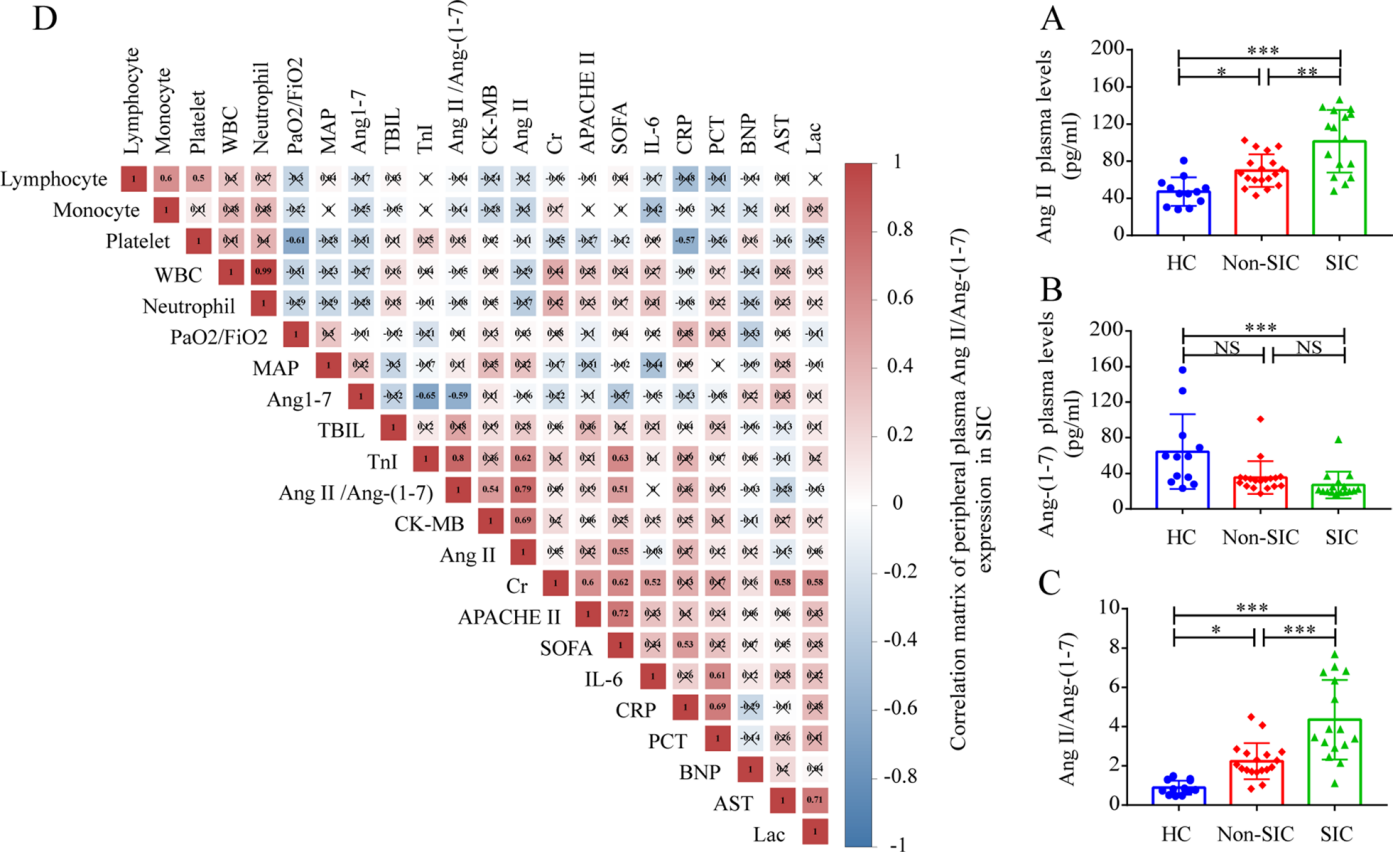
Clinical correlation between plasma Ang II, Ang II/Ang-(1–7), and Ang-(1–7) levels and SIC patients. **A**–**C** Ang-(1–7), Ang II, Ang II/Ang-(1–7), expression levels in the peripheral plasma samples of HC (n = 12), non-SIC (n = 18), and SIC (n = 16) patients. **D** Correlation matrix of plasma Ang-(1–7), Ang II, and Ang II/Ang-(1–7) levels with biochemical indices and clinical scores in the SIC patients (n = 16). The matrix values included the Spearman correlation coefficient (*p* < 0.05). NS: no significant difference, ^*^p < 0.05, ^**^p < 0.01, ^***^p < 0.001

In comparison to non-SIC patients, the SIC patients also showed high BNP, procalcitonin (PCT), lactate, Creatine kinase (CK)-MB, TnI, SOFA, and Acute Physiology and Chronic Health Evaluation (APACHE II) scores. The concentrations of the peripheral plasma Ang II and Ang-(1–7) proteins in different groups were measured using the ELISA method. The findings revealed that peripheral blood Ang II levels were significantly elevated in SIC patients, which were followed by the non-SIC and HC groups (Fig. 1A). However, Ang-(1–7) concentrations were reduced significantly in SIC patients compared to HC patients (Fig. 1B). Additionally, SIC patients showed the highest Ang II/Ang-(1–7) concentration, followed by non-SIC and HC patients (Fig. 1C).

Due to the larger variability in SIC patients, the relationship between clinical markers was also investigated (Fig. 1D). The results indicated that the CK-MB levels (r = 0.69, p = 0.004), SOFA scores (r = 0.55, p = 0.030), and TnI levels (r = 0.62, p = 0.013) were positively and significantly linked with Ang II levels (Fig. 1D). Interestingly, a strong negative relationship was noted between the TnI (r = − 0.65, p = 0.008) and Ang-(1–7) levels (Fig. 1D). Additionally, a positive relationship was observed between Ang II/Ang-(1–7) and CK-MB (r = 0.54, p = 0.034) levels, SOFA score (r = 0.51, p = 0.045), and TnI (r = 0.80, p < 0.001) levels (Fig. 1D). The above clinical data suggested that plasma Ang II/Ang-(1–7), Ang-(1–7), and Ang II could be regarded as potential SIC biomarkers and the SIC-based treatment may be feasible using a targeted RAAS intervention approach.

### Ang-(1–7) alleviates systemic inflammatory response and myocardial injury in SIC mice

**Fig. 2:**
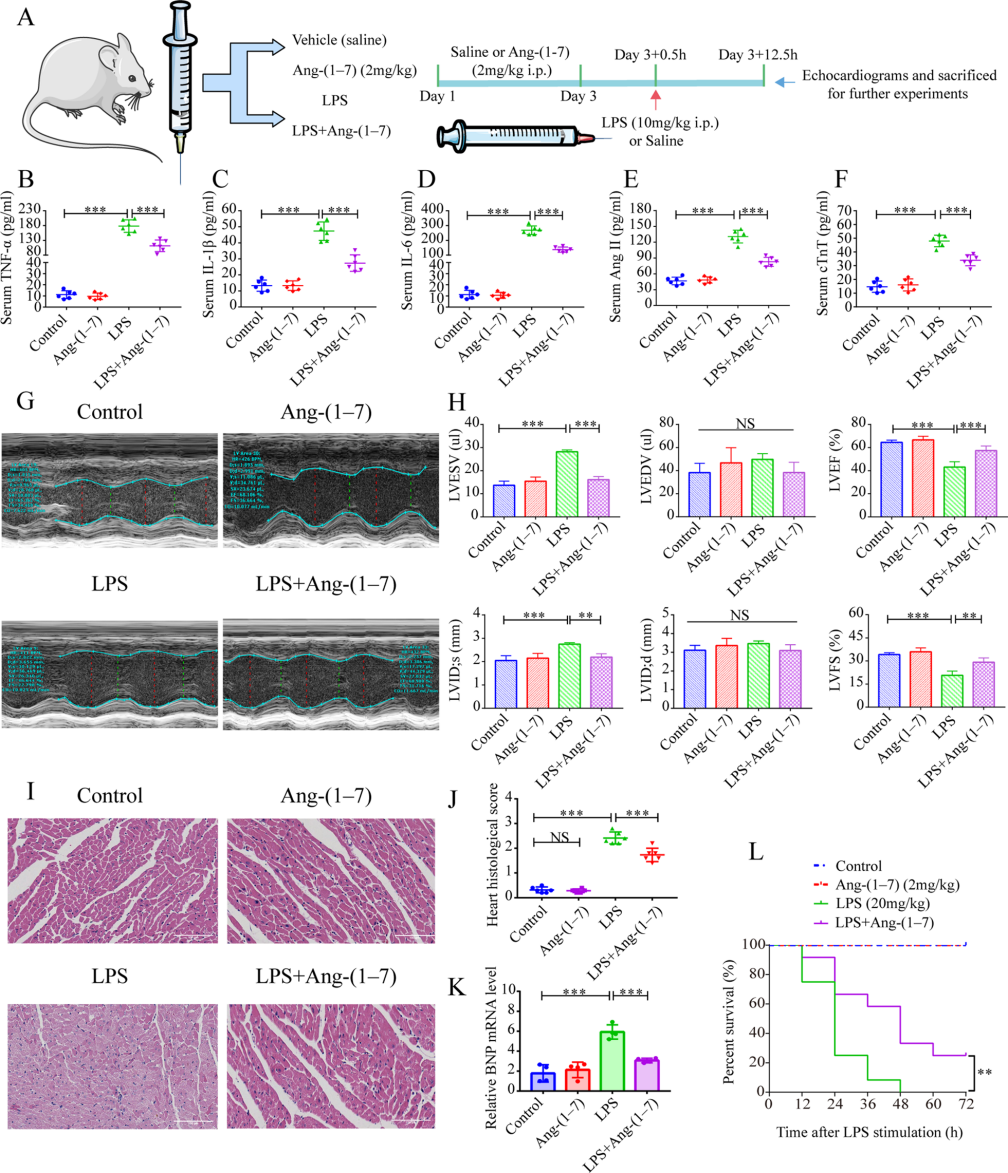
Ang-(1–7) reduces systemic inflammatory response, alleviates myocardial injury, and increases survival in SIC mice. **A** Schematic representation of the animal experiments. Mice were injected with Ang-(1–7) (or same saline volume), IP, daily for 3 days. 30 min after administering the compounds (or saline) for 3 days of the administration, the animals in the corresponding groups were again IP injected by LPS (10 mg/kg) or saline. Echocardiography images were captured 12 h later and mice were sacrificed for further experiments. **B**–**F** Serum Ang II, IL-6, cTnT, IL-1β, and TNF-α levels (n = 6). **G** M-mode echocardiography representative images. **H** Quantitative assessment of LVEF, LVEDV, LVESV, LVID; s, LVID; d, LVFS in every mouse group (n = 4). **I** Representative images of HE staining. **J** Histopathological scores of the heart (n = 6). **K** mRNA levels of BNP in the myocardial samples (n = 4). **L** Influence of Ang-(1–7) pretreatment on the 3^rd^ day of survival of septic mice (n = 12). NS: no significant difference, ^*^p < 0.05, ^**^p < 0.01, ^***^p < 0.001

The regulatory effect of Ang-(1–7), which is an important bioactive product in the RAAS cascade, was investigated on the systemic inflammatory response and cardiac function in SIC mice. Initially, the pretreated mice were subjected to a daily IP injection of Ang-(1–7) for 3 days, and then the LPS (10 mg/kg) IP injection was utilized to construct the SIC model for subsequent experiments (Fig. 2A).

The activation of inflammatory reactions was seen to be the initiating factor in SIC. We found that serum IL-6, TNF-α, and IL-1β concentrations were significantly increased in the LPS group (Fig. 2B–D). Ang-(1–7) pretreatment could reverse the LPS-induced increase of these inflammatory factors (Fig. 2B–D). Additionally, the findings indicated that the serum Ang II level was higher in the LPS group, which could be reversed following Ang-(1–7) pre-treatment (Fig. 2E). All the serum samples collected from the different groups of mice showed similar Ang-(1–7) levels (Additional file 3: Fig. S1A). However, serum Ang II/Ang-(1–7) levels were still higher in the LPS group, which were reversed after Ang-(1–7) pretreatment (Additional file 3: Fig. S1B). These findings indicated that the occurrence of SIC is accompanied by activation of RAAS, which is inhibited by exogenous Ang-(1–7) pretreatment.

The echocardiography images were used to assess the cardiac functions in mice (Fig. 2G). It was noted that the mice in the LPS group developed myocardial dysfunction after 12 h, which manifested as a significant decrease in their Left Ventricular Fractional Shortening (LVFS) and LVEF values, and a significant elevation in their LV end-systolic volume (LVESV) and the LV internal dimension at end-systole (LVID; s) (Fig. 2G–H). Ang-(1–7) pretreatment reversed the LPS-induced myocardial dysfunction, increased the LVFS and LVEF, and decreased LVESV and LVID; s (Fig. 2G–H). Similarly, serum cTnT protein and myocardial BNP mRNA expression levels were elevated in the LPS group, while Ang-(1–7) pretreatment group could significantly reduce these LPS-induced changes (Fig. 2F, K).

The histopathological analysis found that in the Ang-(1–7) and control groups, the tissue structure was complete, the myocardial cells were neatly arranged, and no visible congestion and inflammatory immune cell infiltration were noted (Fig. 2I, J). In the LPS group, the myocardial fibers were disordered, the myocardial tissue was extensively damaged; the outline was unclear, and myocardial lysis and nuclear fragmentation occurred, along with interstitial edema, hyperemia, and inflammatory cell infiltration (Fig. 2I, J). However, LPS-induced myocardial histopathological changes in mice were reversed in Ang-(1–7) pretreatment group (Fig. 2I, J). Also, it was noted that the Ang-(1–7) pretreatment affected the survival of mice after LPS (20 mg/kg) injection (Fig. 2L). The results indicated that the Ang-(1–7) pretreatment group was able to alleviate the lethality caused by LPS compared to the LPS group (Fig. 2L).

### Ang-(1–7) attenuates macrophage infiltration and oxidative stress in the myocardial tissue of SIC mice

**Fig. 3:**
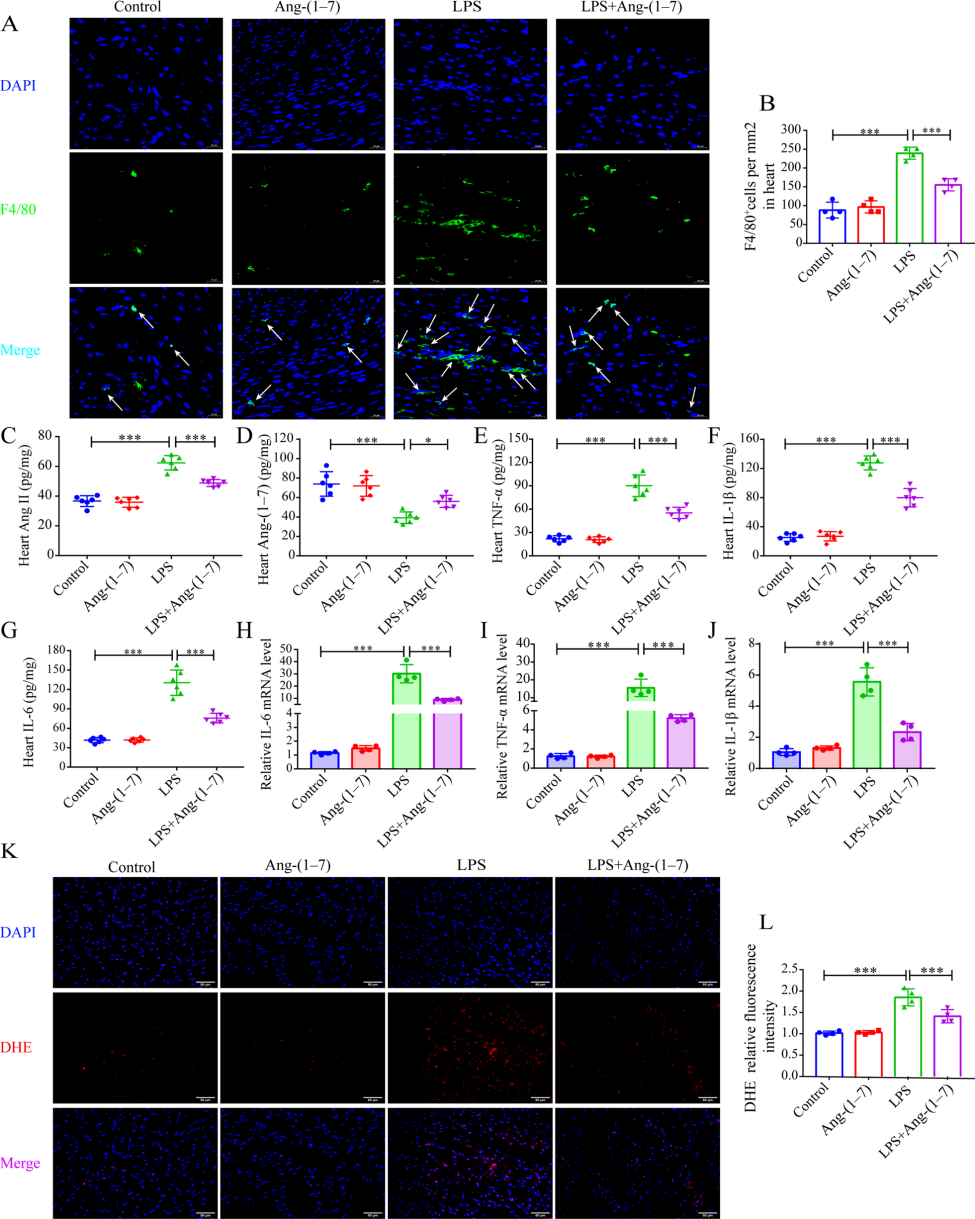
Ang-(1–7) attenuates inflammatory response, macrophage infiltration, and oxidative stress in myocardial tissues of the SIC mice. **A** Representative immunofluorescence images of macrophage infiltration in myocardial tissue of each group (20 μm; × 400, scale bar). **B** Number of F4/80^+^ macrophages (n = 4). **C**–**G** The IL-6, Ang II, TNF-α, Ang-(1–7), and IL-1β protein concentrations in the myocardial specimens (n = 6). **H**–**J** TNF-α, IL-1β, and IL-6 mRNA levels in myocardial tissue samples (n = 4). **K** Images showing the DHE staining in myocardial tissues (50 μm; × 200, scale bar). **L** Relative fluorescence intensity of the DHE procedure (n = 4). ^*^p < 0.05, ^**^p < 0.01, ^***^p < 0.001

Immune cell infiltration, inflammatory reaction, and oxidative stress were regarded as crucial factors affecting the onset and progression of SIC. Hence, the Ang-(1–7) pretreatment effect on SIC mice was investigated in this respect. The findings revealed that the infiltration of macrophages in SIC mice myocardium was increased, whereas the Ang-(1–7) pre-treatment reduced the LPS-induced infiltration of macrophages (Fig. 3A, B). Furthermore, Ang-(1–7) concentration was decreased and Ang II concentration was increased in the myocardial tissues of SIC mice compared to the control group. However, exogenous Ang-(1–7) pretreatment was able to reverse the LPS-induced changes in Ang II and Ang-(1–7) expression in myocardial tissues (Fig. 3C, D). Consistent with these findings, IL-1β, IL-6, TNF-α, protein, and mRNA concentrations were seen to be lower in the myocardial tissue of LPS + Ang-(1–7) mice than LPS mice (Fig. 3E–J). Also, immunofluorescence results showed that LPS increased the oxidative stress in mice myocardial tissues (Fig. 3K, L); however, Ang-(1–7) pretreatment alleviated the LPS-induced increase in oxidative stress (Fig. 3K, L).

### Ang-(1–7) maintains myocardial mitochondrial function and attenuates cardiomyocyte apoptosis in SIC mice

**Fig. 4:**
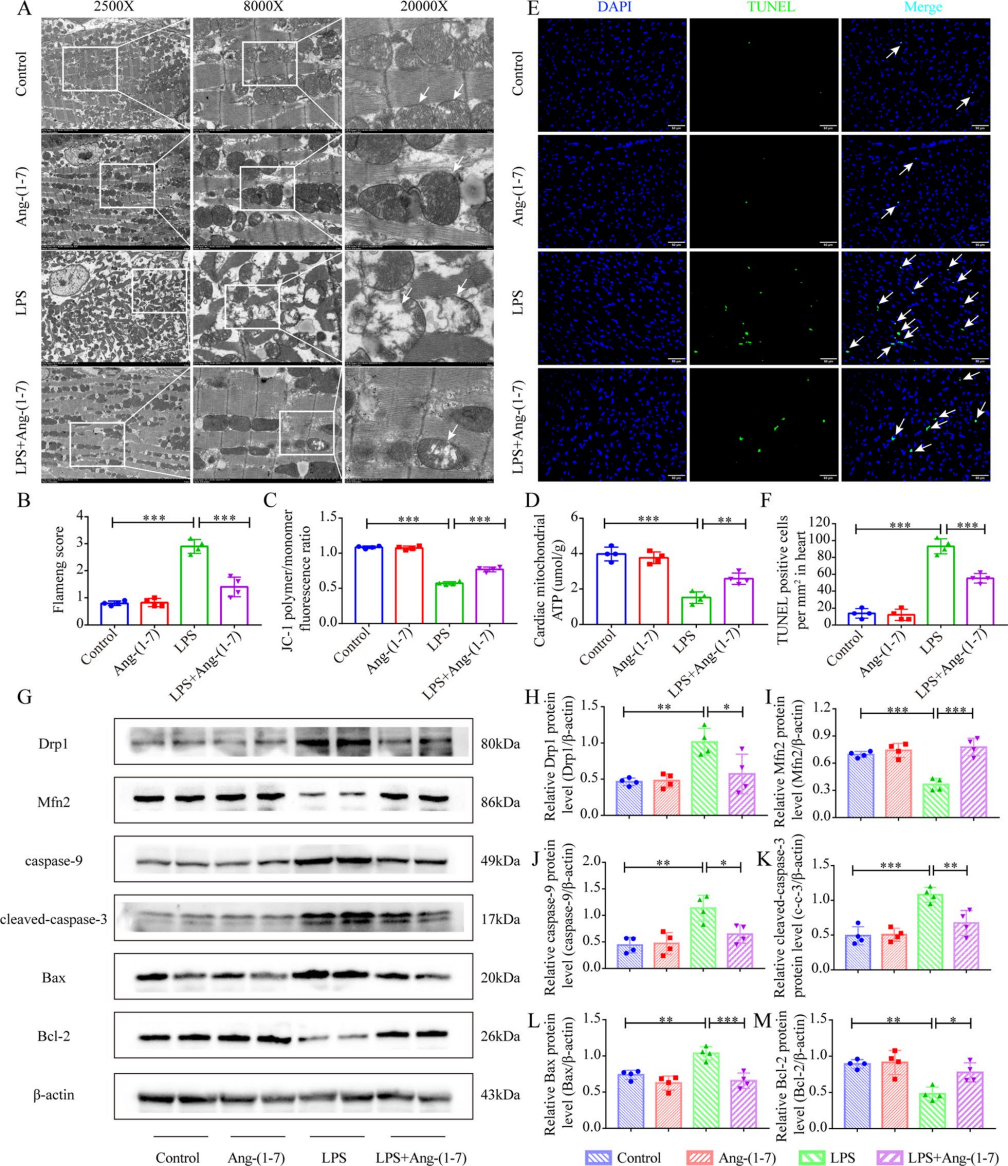
Ang-(1–7) maintains the mitochondrial dynamic equilibrium of cardiomyocytes in SIC mice, reverses their structural dysfunction and damage, and alleviates cardiomyocyte apoptosis. **A** Representative TEM image of myocardial mitochondria. **B** Flameng score of TEM images of mitochondria of cardiomyocytes (n = 4). **C** MMP in myocardial tissue specimens of mice was detected using JC-1 staining (n = 4). **D** Mitochondrial ATP concentration in myocardial tissues (n = 4). **E** TUNEL staining images of the myocardial cellular apoptosis (× 200, scale bar: 50 μm). **F** No. of TUNEL positive cells (n = 4). **G** Western blot images of Bcl-2, cleaved-caspase-3, Bax, Drp1, Mfn2, and caspase-9 in myocardial tissues. (**H**–**M**) Quantification of the caspase-9, Drp1, cleaved-caspase-3, Bcl -2, Mfn2, and Bax proteins concentrations in myocardial specimens (n = 4). ^*^p < 0.05, ^**^p < 0.01, ^***^p < 0.001

Mitochondria are the main organelles that supply energy to the cardiomyocytes. Hence, the altered mitochondrial structure and dysfunction can lead to aggravated cardiomyocyte damage. TEM analysis indicated that LPS caused myocardial fiber lysis, severe mitochondrial swelling, mitochondrial cristae loss, and vacuolar degeneration in mice (Fig. 4A, B). Ang-(1–7) pretreatment can reverse myofibrolysis, and reduce the mitochondrial cristae damage and mitochondrial swelling (Fig. 4A, B). Damage to the mitochondrial structure will affect MMP, resulting in abnormal oxidative phosphorylation and reduced ATP production. The results suggested that LPS reduced the ATP and MMP levels in mouse myocardial tissues, but Ang-(1–7) pretreatment reversed the LPS-induced reduction. (Fig. 4C, D).

Mitochondrial damage and changes in energy metabolism will break the homeostasis of mitochondrial fusion and fission and continuously aggravate the damage. The findings of the western blotting technique implied that mitofusin 2 (Mfn2) protein concentration was decreased, while dynamin-related protein 1 (Drp1) protein concentration was significantly increased in myocardial tissue samples of LPS-induced SIC mice (Fig. 4G–I). Additionally, the cleaved caspase-3, Bcl2-associated X protein (Bax), and caspase-9 protein concentrations were elevated, whereas the B cell lymphoma 2 (Bcl-2) protein levels were lowered in SIC mice (Fig. 4G, J–M). However, Ang-(1–7) pretreatment could eliminate all these changes (Fig. 4G–M). Similarly, the TUNEL assay was carried out and a significant elevation was noted in the TUNEL-positive cells in myocardial tissues of LPS-induced SIC mice, and Ang-(1–7) pretreatment could reverse this phenomenon (Fig. 4E, F).

### Ang-(1–7) inhibits NF-κB and MAPK pathway activation in the myocardial tissue of SIC mice

**Fig. 5:**
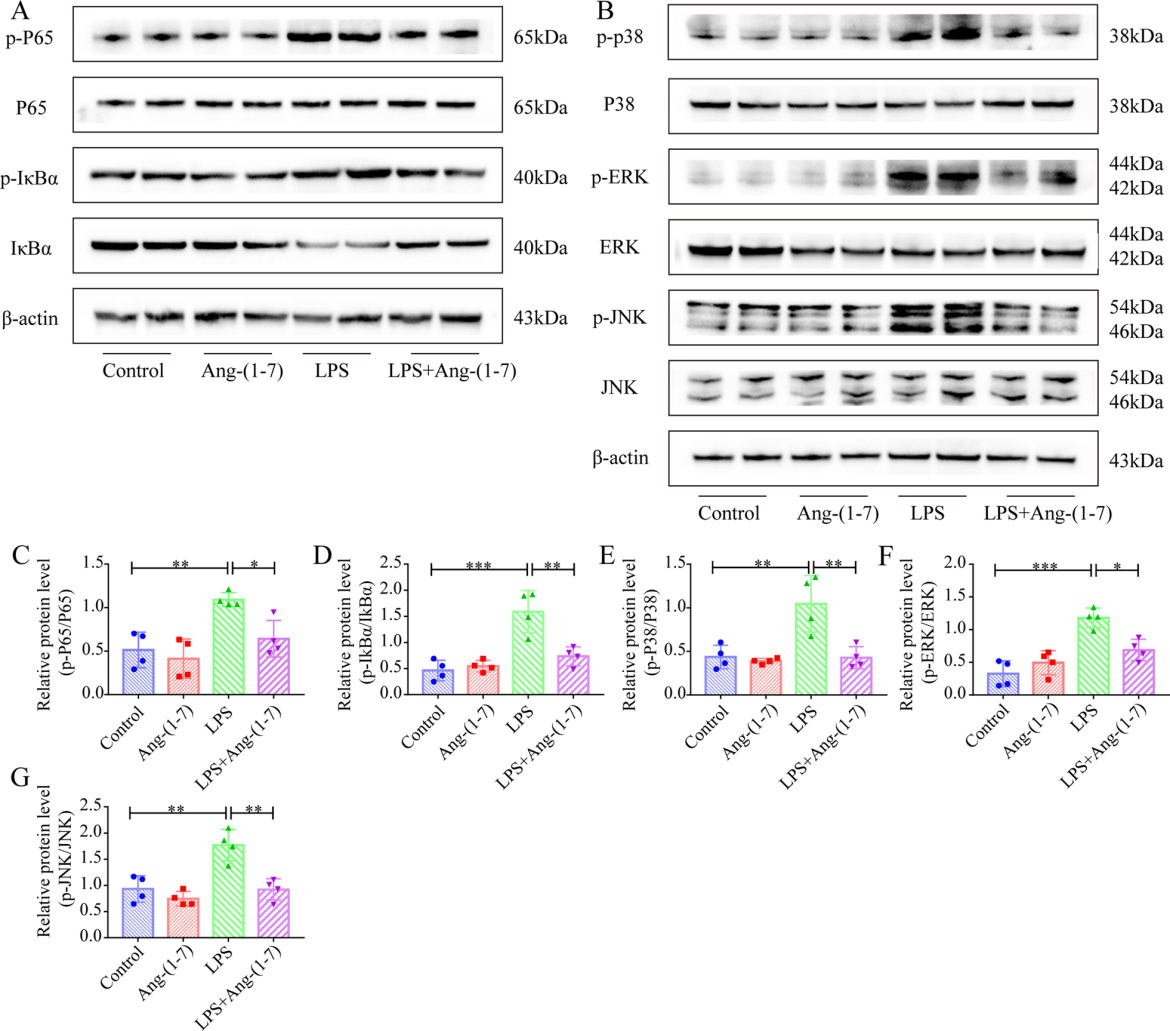
Ang-(1–7) suppresses the upregulation of the NF-κB and MAPK pathways in the myocardial tissues of SIC mice. **A** Western blot images of p-IκBα, IκBα, p-P65, and P65 in the myocardial specimens. **B** Western blot images of p-P38, p-JNK, JNK, p-ERK, P38, ERK in the myocardial tissues. **C**–**G** Quantifying the p-P38/P38, p-IκBα/IκBα, p-ERK/ERK, p-P65/P65, and p-JNK/JNK concentrations in myocardial tissues (n = 4). ^*^p < 0.05, ^**^p < 0.01, ^***^p < 0.001

The activation of the NF-κB and MAPK signaling pathways was involved in the occurrence of the inflammatory storm in SIC mice. In this study, the protective role played by Ang-(1–7) in reducing inflammatory and mitochondrial damage in SIC murine myocardium tissues and the effect of Ang-(1–7) on the NF-κB and MAPK signaling pathways were examined. The findings revealed that the P38, c-Jun N-terminal kinase (JNK), extracellular signal-regulated kinase (ERK), IκBα, and P65 phosphorylation levels were upregulated significantly in the myocardial tissue of the LPS-induced SIC mice (Fig. 5A–G). However, Ang-(1–7) pretreatment could reverse the above LPS-induced changes (Fig. 5A–G). Additionally, the results also showed the LPS-induced increase in p65 (nucleus) protein expression levels in the myocardial tissue of SIC mice (Additional file 3: Fig. S2A). However, pretreatment with Ang-(1–7) could inhibit the nuclear translocation of p65, induced by LPS (Additional file 3: Fig. S2A).

### Ang-(1–7) inhibits oxidative stress, inflammatory response, and mitochondrial dysfunction in the LPS-induced H9c2 cells

**Fig. 6:**
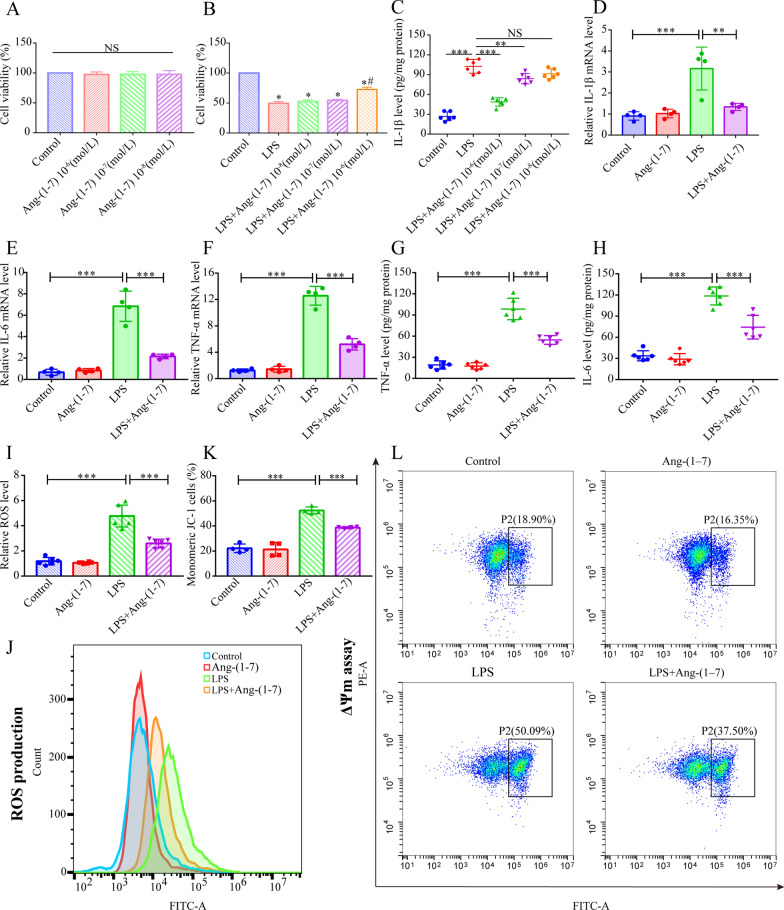
Ang-(1–7) inhibits inflammatory response, mitochondrial dysfunctioning, and oxidative stress, in LPS-induced H9c2 cells. **A** The H9c2 cell viability, after being pretreated with varying concentrations of Ang-(1–7) (10^–6^, 10^–7^, 10^–8^ mol/L) for 12 h, was measured using the CCK8 assay (n = 6). **B** The H9c2 cell viability after Ang-(1–7) (10^–6^, 10^–7^, 10^–8^ mol/L) pretreatment for 1 h before LPS stimulation for 12 h was measured using the CCK8 assay (n = 6). **C** IL-1β protein concentration in the H9c2 cells (n = 6). **D**–**F** The IL-1β, TNF-α, and IL-6 mRNA expression levels in H9c2 cells (n = 4). **G**, **H** TNF-α and IL-6 protein concentrations in H9c2 cells (n = 6). **I**, **J** Quantitative analysis and flow cytometry histograms depicting the ROS production in H9c2 cells (n = 6). **K**, **L** Quantitative analysis and flow cytometry plots describing the ratio of monomer JC-1 cells (representing mitochondrial damage) in every group (n = 4). Cells with monomeric JC-1 were green in the P2 region, representing damaged mitochondria. **B** ^*^p < 0.05 than control, ^#^*p* < 0.05 than LPS mice. **D**–**L** Ang-(1–7) intervention using the pretreatment concentration of 10^–6^ mol/L for 1 h, and LPS activation of 1 ug/mL for 12 h. NS: no significant difference, ^*^p < 0.05, ^**^p < 0.01, ^***^p < 0.001

The toxicity of Ang-(1–7) was tested at different concentrations on the H9c2 cells and its pretreatment influence on the viability of LPS-stimulated H9c2 cells was estimated with the CCK-8 assay (Fig. 6A, B). The findings indicated that all tested concentrations of Ang-(1–7) were nontoxic to H9c2 cells (Fig. 6A). H9c2 cells were preincubated with varying Ang-(1–7) protein concentrations for 60 min before LPS stimulation, and the H9c2 cell viability increased in a dose-dependent technique with an elevation in the Ang-(1–7) concentrations (Fig. 6B). Furthermore, ELISA indicated that the Ang-(1–7) levels of 10^−6^ mol/L could significantly inhibit the IL-1β expression in H9c2 cells (Fig. 6C). Therefore, Ang-(1–7) (at 10^−6^ mol/L) could be used for pretreatment in the following experiments.

When stimulated by LPS, the IL-1β, TNF-α, and IL-6, mRNA levels, and the IL-6 and TNF-α protein levels increased in H9c2 cells, but Ang-(1–7) pretreatment could eliminate this change (Fig. 6D–H). Excessive pro-inflammatory factors exacerbate oxidative stress and mitochondrial damage in H9c2 cells. We observed that LPS increased ROS production and JC-1 monomer proportion (representing mitochondrial damage) in H9c2 cells (Fig. 6I–L). However, Ang-(1–7) pretreatment could decrease the excess ROS and stabilize the MMP in H9C2 cells in contrast with the LPS group (Fig. 6I–L).

### Ang-(1–7) maintains mitochondrial dynamic equilibrium in H9c2 cardiomyocytes and alleviates LPS-induced apoptosis

**Fig. 7:**
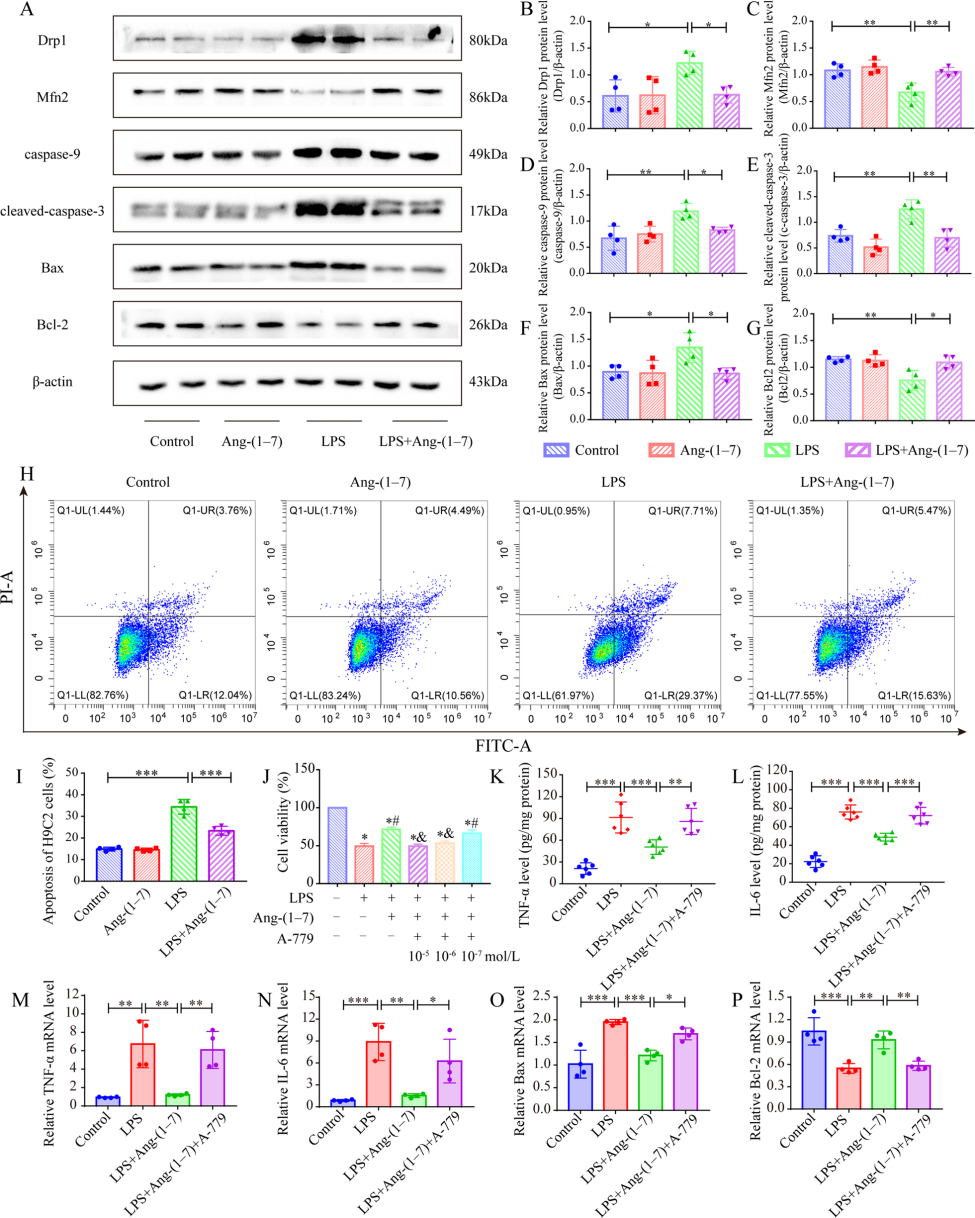
Ang-(1–7) maintained mitochondrial dynamic equilibrium in H9c2 cardiomyocytes and alleviated LPS-induced apoptosis. **A** Western blot images of Drp1, Mfn2, caspase-9, cleaved caspase-3, Bcl-2, and Bax in H9c2 cells. **B**–**G** Quantification of the concentrations of cleaved caspase-3, Drp1, Mfn2, Bcl-2, caspase-9, and Bax proteins in H9c2 cells (n = 4). **H**, **I** Flow cytometry plots and quantitative analysis depicting the proportion of H9c2 apoptotic cells (n = 4). **J** Before LPS stimulation of H9c2 cells for 12 h, cells in each group were pretreated using differing concentrations of A-779 (10^–5^,10^–6^,10^–7^ mol/L) for 2 h, using Ang-(1–7) (10^–6^ mol/L) for 1 h and then with CCK8 assay for cell viability (n = 6). ^*^p < 0.05 than the control group, ^#^*p* < 0.05 than LPS group, ^&^*p* < 0.05 in comparison to LPS + Ang-(1–7) group. **K**, **L**) The IL-6 and TNF-α protein concentrations in H9c2 cells (n = 6). **M**–**P** IL-6, Bcl-2, TNF-α, and the Bax mRNA levels in the H9c2 cells (n = 4). **K**–**P** The A-779 intervention was pretreatment with 10^–5^ mol/L for 2 h, the Ang-(1–7) intervention included 10^–6^ mol/L pretreatment for 1 h, and LPS activation of 1 µg/mL for 12 h. ^*^p < 0.05, ^**^p < 0.01, ^***^p < 0.001

Furthermore, mitochondrial dynamics-related proteins and mitochondria-dependent apoptosis-related protein levels in H9c2 cells were estimated with the help of the Western blot technique. The findings revealed that LPS disrupted the mitochondrial dynamic balance in the H9c2 cells, increased the Drp1 protein concentration, and decreased the Mfn2 protein concentration (Fig. 7A–C). Additionally, LPS also elevated the Bax, caspase-9, and cleaved caspase-3 protein levels, but lowered the Bcl-2 protein level in H9c2 cells (Fig. 7A, D–G). However, Ang-(1–7) pretreatment can reverse the above changes (Fig. 7A, D–G). Similarly, it was also seen that LPS increased the apoptosis rate of H9c2 cells, but Ang-(1–7) pretreatment could significantly reverse this phenomenon (Fig. 7H, I).

Also, to determine if Ang-(1–7) alleviates LPS-induced H9c2 cell damage through its functional Mas receptor (MasR), the MasR antagonist A-779 was utilized in the subsequent experiments. Firstly, the effect of various concentrations of A-779 pretreatment on the viability of H9c2 cells preincubated in the presence of Ang-(1–7), and stimulated with LPS, was evaluated by CCK-8 assay. It was noted that Ang-(1–7) pretreatment could improve the viability of H9c2 cells compared to the LPS group (Fig. 7J). However, the repressive function of Ang-(1–7) on the LPS-induced reduction of H9c2 cell activity could be reversed when pretreated with A-779 (10^−5^ mol/L) (Fig. 7J). Therefore, A-779 (10^−5^ mol/L concentration) was used for pretreatment in subsequent experiments. Further experiments showed that A-779 could reverse the inhibitory effect of the Ang-(1–7) on LPS-stimulated changes of IL-6, TNF-α, Bcl2, and Bax transcription or/and protein levels in H9c2 cells (Fig. 7K–P). In conclusion, Ang-(1–7) functions mainly through MasR, and A-779 can reverse the protective impact of Ang-(1–7) on the inflammatory response and H9c2 cell apoptosis.

### Ang-(1–7) inhibits the LPS-induced stimulation of NF-κB and MAPK pathway in H9c2 cardiomyocytes

**Fig. 8:**
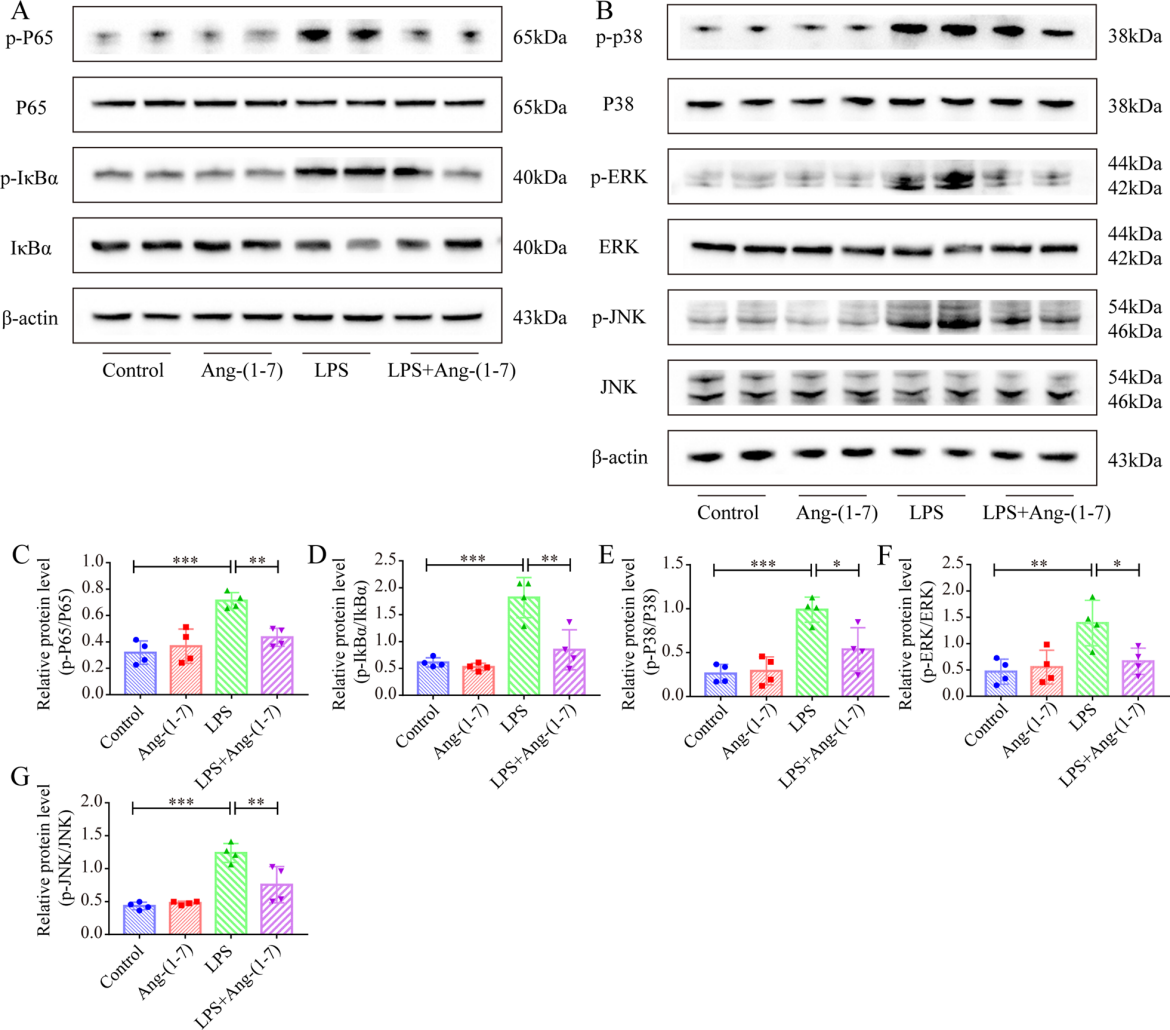
Ang-(1–7) inhibits LPS-induced activation of NF-κb and MAPK pathways in H9c2 cardiomyocytes. **A** Western blotting images of the p-P65, p-IκBα, P65, and IκBα in H9c2 cells. **B** Western blots of p-JNK, p-P38, p-ERK, P38, ERK, and JNK in H9c2 cells. **C**–**G** Quantifying the concentrations of p-IκBα/IκBα, p-ERK/ERK, p-JNK/JNK, p-P38/P38, and p-P65/P65 proteins in H9c2 cells (n = 4). ^***^p < 0.001, ^**^p < 0.01, ^*^p < 0.05

In this study, experiments were carried out to determine if Ang-(1–7) affected the LPS-stimulated proinflammatory cytokine expression and apoptosis in H9c2 cells by controlling NF-κB and MAPK pathways. Initially, the subcellular translocation of NF-κB was investigated and the findings revealed that the p65 expression in the nucleus was significantly enhanced in the LPS-induced H9c2 group (Additional file 3: Fig. S2B, C). However, Ang-(1–7) pretreatment alleviated p65 nuclear translocation induced by LPS (Additional file 3: Fig. S2B, C). On the other hand, the results revealed that LPS activated NF-κb and MAPK pathways in H9c2 cells, and significantly upregulated the JNK, P38, iκBα, ERK, and P65 protein phosphorylation levels (Fig. 8A–G). As expected, Ang-(1–7) pretreatment significantly reduced the LPS-induced elevation in the phosphorylation levels of the above proteins (Fig. 8A–G). In addition, different pathway inhibitors (p38 inhibitor SB202190, ERK inhibitor U0126, JNK inhibitor SP600125, NF-κB inhibitor TPCK and TLR4 inhibitor TAK-242) were used to further validate the causal relationship between NF-κB and MAPK pathways and Ang-(1–7) regulation of SIC in vitro models. The results showed that these inhibitors were able to reverse the LPS-induced increase in IL-1β, IL-6, and Bax protein levels as well as the decrease in Bcl-2 protein levels in H9c2 cells (Additional file 3: Fig. S3A–E). Furthermore, the IL-1β, IL-6, Bax and Bcl-2 protein levels were similar in the Ang-(1–7) + inhibitor group and Ang-(1–7) only pretreatment group.

(Additional file 3: Fig. S3A–E).Taken together, Ang-(1–7) pretreatment can protect H9c2 cells from LPS-induced damage by regulating NF-κb and MAPK pathways.

## Discussion

This study has investigated the involvement of Ang (1–7) in SIC and determined whether it could be used as an effective treatment strategy for SIC patients. SIC can be described as cardiac dysfunction owing to sepsis, affecting many patients in the Intensive care units (ICUs) [2]. The results in this study indicated that the SIC patients showed significantly higher peripheral plasma Ang II and Ang II/Ang-(1–7) levels than those expressed in HC and non-SIC patients, but Ang-(1–7) expression was lowered significantly compared to those expressed in HC. Peripheral plasma Ang II, Ang II/Ang-(1–7), and Ang-(1–7) levels in SIC patients were significantly related to the level of myocardial injuries. The findings indicate that the RAAS activation in SIC can lead to a homeostasis imbalance between the Ang-(1–7)/MasR and Ang II/AT1 axis. Plasma Ang II, Ang II/Ang-(1–7), and Ang-(1–7) concentrations could be regarded as potential biomarkers of SIC. To further investigate and validate the hypothesis, the influence and mechanisms of exogenous Ang-(1–7) in the in vivo and in vitro SIC models were tested. The results show that Ang-(1–7) regulates the inflammatory response, reduces oxidative stress, maintains mitochondrial homeostasis, and alleviates the mitochondrial structure and function damage through NF-κb and MAPK pathways, thereby alleviating SIC. The findings revealed that Ang-(1–7) could be regarded as an efficient therapeutic drug for SIC.

There are no available efficient treatment plans for SIC patients in clinic settings, who are in critical condition and have a significant fatality rate [7]. Compared to non-SIC patients, SIC patients showed higher SOFA and APACHE II scores, as well as higher levels of CK-MB, BNP, TnI, lactate, and PCT. This conclusion was consistent with those presented in earlier studies [48–52]. RAAS was stimulated and the Ang II levels are elevated during the initial sepsis stages [53]. In this study, the results indicated that the SIC patients showed higher Ang II and Ang II/Ang-(1–7) levels, followed by non-SIC patients and HC. Additionally, SIC patients had low Ang-(1–7) levels than HC patients. Relevant studies indicated that RAAS was activated in COVID-19 patients, which led to an increase in their peripheral plasma Ang II concentrations, whereas their Ang-(1–7) concentration was decreased [54]. Additionally, the d-dimer and proinflammatory cytokines showed a negative relationship with Ang-(1–7) expression levels [54]. In COVID-19 patients, lower Ang-(1–7) levels influenced the activation of multiple immunological responses and disseminated coagulation [54]. Furthermore, the COVID-19 patients, who died, had lower serum Ang-(1–7) levels and correspondingly higher Ang II/Ang-(1–7) levels [55]. Thus, the Ang II/Ang-(1–7) concentrations could be regarded as independent predictors of COVID-19 mortality [55]. This result offers convincing evidence that Ang II and Ang-(1–7) could play a role in SIC. It also suggests that using drugs to target the regulation of RAAS homeostasis may be effective in treating SIC patients.

During the occurrence of the sepsis-induced organ injury, RAAS is often activated, while the angiotensin-ACE2/Ang-(1–7)/MasR axis gets down-regulated, and the ACE/Ang II/AT1R axis is up-regulated [56]. Sepsis-induced ALI causes an imbalance in the ACE2/ACE and AngII/Ang-(1–7), and the ACE2 overexpression can up-regulate ACE2/Ang-(1–7)/MasR axis to relieve ALI [37, 39]. Studies revealed that targeted inhibition of midkine expression in the lung samples suppresses ACE activity through Notch 2 receptors and decreases the secretion of angiotensin II, thereby improving sepsis-induced ALI [57]. Exogenous Ang-(1–7) can significantly alleviate SIAKI by reducing oxidative stress and inflammation in renal tissue [41]. Furthermore, lipoxin receptor agonists (BML-11) also can alleviate LPS-induced lung and liver injury by modulating the RAAS [58]. Notably, neuromodulin-1 (NRG-1), adenosine, and Chinese yam can reduce the inflammatory response, protect cardiomyocytes, and ameliorate sepsis-induced cardiac dysfunction by inhibiting RAAS overactivation [59, 60]. Experiments were carried out in this study to determine if the exogenous Ang-(1–7) pretreatment could regulate RAAS, and the outcome of these experiments showed that Ang-(1–7) effectively inhibits the biological function of Ang II, thereby alleviating SIC. Excessive inflammatory responses and oxidative stress can aggravate cardiomyocyte apoptosis and promote the occurrence and development of SIC [61, 62]. We observed that the LPS stimulation resulted in an excessive inflammatory response, elevated ROS production, and increased cardiomyocyte apoptosis in the in vivo and in vitro SIC models. However, this phenomenon could be reversed by Ang-(1–7) pretreatment. An earlier study stated that Ang-(1–7) inhibits the infiltration of splenic macrophages, and also regulates the in vitro and in vivo polarization of macrophages to reduce the inflammatory injury of sepsis [43]. Similarly, Souza et al. found that the Ang-(1–7) also inhibited the inflammatory responses and prevented hypothermia in septic mice [63]. Increased RAAS activity in sepsis can lead to endothelial dysfunction and aggravated oxidative stress, and pretreatment with Ang-(1–7) can reduce lipid peroxidation, superoxide, and ROS generation [16, 64]. Related studies have shown that exogenous Ang-(1–7) intervention can attenuate pulmonary and hepatic fibrosis by inhibiting oxidative stress [20, 28]. In addition, exogenous Ang-(1–7) pretreatment can also attenuate doxorubicin-mediated oxidative stress, thereby improving cardiac dysfunction [65]. Furthermore, a higher apoptosis was noted in the organ injuries caused by severe sepsis, and inhibition of apoptosis could improve the survival rate of septic animal models [16, 66]. Studies implied that the Ang-(1–7) inhibits the LPS and Ang II-induced apoptosis in epithelial and endothelial cells, as well as the apoptosis of mouse hepatocytes induced by sepsis [16, 39, 67, 68]. These results implied that Ang-(1–7) can regulate RAAS and exert its anti-apoptotic, antioxidant, and anti-inflammatory effects, which were consistent with the results presented in this study.

Earlier studies have found that the energy supply of cardiomyocytes mainly depends on mitochondria, and the structure and function of mitochondria in cardiomyocytes will be abnormal when SIC occurs [61, 69, 70]. In this study, the mitochondrial dynamics of SIC mice were disturbed, which was related to the excessive inflammatory responses, the lower energy production of cardiomyocytes, and increased oxidative stress, wherein all these factors interacted with each other to eventually increase the rate of cardiomyocyte apoptosis. Notably, it was seen that Ang-(1–7) can alleviate mitochondrial dysfunctioning and inhibit the apoptosis of cardiomyocytes by regulating LPS-induced abnormal RAAS activation and inflammatory response according to in vivo and in vitro experiments. The Ang-(1–7) mainly wields its effect through MasR, and the Ang-(1–7) protective function on sepsis can be eliminated by initially injecting the septic mice with A-779, a MasR-specific inhibitor [43, 56]. To confirm the fact that Ang-(1–7) exerts its role via the Ang-(1–7)/MasR axis, in vitro experiments were conducted in this study using A-779, and the findings implied that A-779 could reverse the Ang-(1–7) protective effect on H9c2 cell inflammatory response and apoptosis.

LPS activated the NF-κB and MAPK pathways and induced inflammatory responses [71–74]. The relationship between RAAS and NF-κB inflammatory factors is complex and interrelated. Similarly, higher Ang II levels combined with AT1R under abnormal activation of RAAS can also activate the NF-κB signaling pathway to generate many inflammatory cytokines [75, 76]. Related studies showed that Ang-(1–7) can improve sepsis-induced organ injury by controlling the NF-κB and MAPK pathway [41, 43, 56]. Also, some researchers noted that Ang-(1–7) can improve cardiovascular diseases by controlling the MAPK pathway [77–80]. The findings in this study indicated that Ang-(1–7) can relieve SIC by regulating NF-κB and MAPK pathways during the in vivo and in vitro experiments. Thus, the findings revealed that RAAS could be upstream of NF-κB and MAPK pathways, and the impact of Ang-(1–7) for SIC alleviation may be mediated by regulating NF-κB and MAPK pathways.

There are some limitations in this study. Firstly, the relatively small sample size of patients included in our clinical study did not lend itself to multivariate analysis. Secondly, the influence of disease severity and volume status on plasma Ang-(1–7) and Ang II variation could not be excluded. Large-scale clinical studies need to be conducted in the future to further validate the clinical significance of Ang-(1–7) and Ang II in SIC patients. Finally, in vitro and in vivo experiments indicated that Ang-(1–7) could be used as a potential therapeutic target for SIC, however, this strategy needs to be assessed in patients in the future.

## Conclusions

**Fig. 9:**
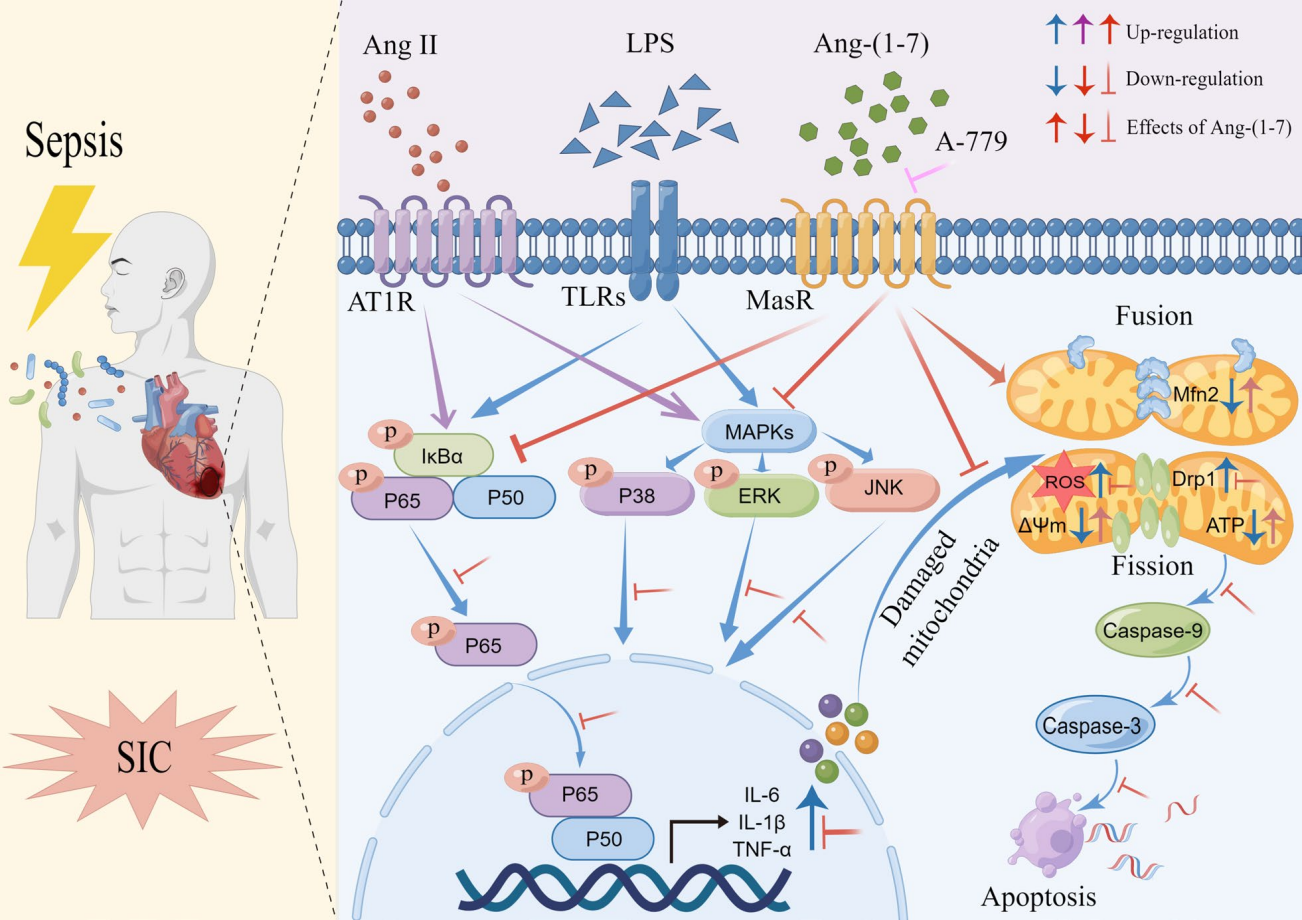
Schematic diagram showing the protective effect of Ang-(1–7) on SIC mice and its underlying mechanism. LPS can induce RAAS activation and increase Ang II secretion in blood, tissues, and cells. LPS and Ang II can activate NF-κB and MAPK pathways and generate a large number of inflammatory mediators. Excessive production of proinflammatory factors aggravates oxidative stress, mitochondrial damage, and dynamic imbalance in cardiomyocytes, and further leads to increased apoptosis of cardiomyocytes. Ang-(1–7) may regulate inflammation, reduce oxidative stress, maintain mitochondrial homeostasis, and alleviate mitochondrial structural and functional damage through NF-κB and MAPK pathways

In conclusion, this study observed that the plasma Ang II, Ang II/Ang-(1–7), and Ang-(1–7) levels were useful biomarkers among patients suffering from SIC. Additionally, it was established that Ang-(1–7) regulates the oxidative stress, inflammatory responses, and mitochondrial dynamic equilibrium through NF-κB and MAPK pathways to alleviate SIC (Fig. 9), indicating that it could be used as a potential therapeutic strategy for SIC.

## Supplementary Information

**Additional file 1: Table S1.** Primers for quantitative real-time PCR.

**Additional file 2: Table S2.** Demographic and clinical parameters of the study population.

**Additional file 3: Fig S1.** Ang-(1–7) and Ang II/Ang-(1–7) levels in the serum of each group of mice. **Fig S2.** Effect of Ang-(1–7) on LPS-mediated NF-κB nuclear translocation. **Fig S3.** Ang-(1–7) alleviates H9c2 cells apoptosis induced by LPS-mediated inflammatory response through the NF-κB and MAPK signaling pathways.

## Data Availability

Any data involved in this study can be requested from the corresponding author.
