# Supplementary material for: Angiotensin-(1–7) ameliorates sepsis-induced cardiomyopathy by alleviating inflammatory response and mitochondrial damage through the NF-κB and MAPK pathways
Source: J Transl Med. 2023 Jan 2;21:2. doi: 10.1186/s12967-022-03842-5 (PMC9807106; doi:10.1186/s12967-022-03842-5)
Supplement: Supplementary file 1 — Additional file 1: Table S1. Primers for quantitative real-time PCR. [file 12967_2022_3842_MOESM1_ESM.docx]

Table S1: Primers for quantitative real-time PCR.

| **Gene** | **Forward primer (5'–3')** | **Reverse primer (5'–3')** |
| --- | --- | --- |
| Mouse TNF-α | CCCTCACACTCAGATCATCTTCT | GCTACGACGTGGGCTACAG |
| Mouse IL-6 | TAGTCCTTCCTACCCCAATTTCC | TTGGTCCTTAGCCACTCCTTC |
| Mouse IL-1β | GCAACTGTTCCTGAACTCAACT | ATCTTTTGGGGTCCGTCAACT |
| Mouse BNP | GAGGTCACTCCTATCCTCTGG | GCCATTTCCTCCGACTTTTCT |
| Mouse GAPDH | AGGTCGGTGTGAACGGATTTG | TGTAGACCATGTAGTTGAGGTCA |
| Rat IL-1β | TGTGGCAGCTACCTATGTCT | GGGAACATCACACACTAGCA |
| Rat TNF-α | CGTGTTCATCCGTTCTCTACC | GCAATCCAGGCCACTACTT |
| Rat IL-6 | CCGTTTCTACCTGGAGTTTGT | GTTTGCCGAGTAGACCTCATAG |
| Rat BAX | CAGGGTTTCATCCAGGATCGAGCAGG | CGGGGGGAGTCCGTGTCCACGTCAG |
| Rat Bcl-2 | CCAGCGTGTGTGTGCAAGTGTAAAT | ATGTCAATCCGTAGGAATCCCAACC |
| Rat GAPDH | GCACCGTCAA GCTGAGAAC | TGGTGAAGACGCCAGTGGA |
