# Supplementary material for: Angiotensin-(1–7) ameliorates sepsis-induced cardiomyopathy by alleviating inflammatory response and mitochondrial damage through the NF-κB and MAPK pathways
Source: J Transl Med. 2023 Jan 2;21:2. doi: 10.1186/s12967-022-03842-5 (PMC9807106; doi:10.1186/s12967-022-03842-5)
Supplement: Supplementary file 2 — Additional file 2: Table S2. Demographic and clinical parameters of the study population. [file 12967_2022_3842_MOESM2_ESM.docx]

Table S2: Demographic and clinical parameters of the study population.

| Variables | HC (n = 12) | Non-SIC (n = 18) | SIC (n = 16) | p |
| --- | --- | --- | --- | --- |
| **Baseline characteristics** |  |  |  |  |
| Male [n (%)] | 6 (50.00) | 8 (44.44) | 9 (56.25) | 0.790 |
| Age (years) | 57.67 ± 9.58 | 65.06 ± 14.28 | 67.06 ± 15.32 | 0.186 |
| COPD [n (%)] | NA | 6 (33.33) | 3 (18.75) | 0.448 |
| Diabetes mellitus [n (%)] | NA | 9 (50.00) | 5 (31.25) | 0.315 |
| Hypertension [n (%)] | NA | 13 (72.22) | 9 (56.25) | 0.475 |
| Coronary heart disease [n (%)] | NA | 6 (33.33) | 9 (56.25) | 0.300 |
| **Source of sepsis** |  |  |  | 0.501 |
| Lung [n (%)] | NA | 9 (50.00) | 8 (50.00) |  |
| Alimentary tract [n (%)] | NA | 2 (11.11) | 2 (12.50) |  |
| Urinary tract [n (%)] | NA | 6 (33.33) | 4 (25.00) |  |
| Cranial cavity [n (%)] | NA | 1 (5.56) | 0 |  |
| Skin soft tissue [n (%)] | NA | 0 | 2 (12.50) |  |
| **Severity score** |  |  |  |  |
| SOFA | NA | 5 (4-6) | 10 (8-12) ^†^ | <0.001 |
| APACHE II | NA | 16 (14-17) | 20 (18-22) ^†^ | 0.002 |
| **Laboratory data** |  |  |  |  |
| PaO_2_/FiO_2_ | NA | 244 (207-332) | 255 (178-270) | 0.427 |
| MAP (mmHg) | NA | 71.56 ± 3.11 | 70.69 ± 3.11 | 0.423 |
| TBIL(μmol/L) | 14.9 (12.9-17.0) | 27.0 (15.5-118.5) ^*^ | 61 (29.3-151.3) ^*^ | <0.001 |
| WBC | 5.78 ± 1.20 | 13.58 ± 7.76^*^ | 14.45 ± 9.59^*^ | 0.008 |
| Neutrophil count (×10^9^/L) | 3.40 ± 0.94 | 12.12 ± 7.37^*^ | 13.10 ± 9.33^*^ | 0.002 |
| Lymphocyte count (×10^9^/L) | 1.71 (1.48-2.17) | 0.51 (0.29-0.90) ^*^ | 0.84 (0.46-1.28) ^*^ | <0.001 |
| Monocyte count (×10^9^/L) | 0.40 (0.37-0.47) | 0.52 (0.22-1.03) | 0.52 (0.22-0.71) | 0.663 |
| Platelet counts (×10^9^/L) | 202 (155-252) | 102 (76-178) ^*^ | 98 (33-193) ^*^ | 0.005 |
| Creatinine (μmol/L) | 56 (45-77) | 100 (61-209) ^*^ | 153 (75-265) ^*^ | 0.004 |
| AST (U/L) | 20 (15-27) | 40 (22-81) ^*^ | 89 (43-183) ^*^ | <0.001 |
| IL-6 (pg/mL) | NA | 73 (30-274) | 124 (44-587) | 0.204 |
| CRP (mg/L) | NA | 74 (26-140) | 123 (44-176) | 0.201 |
| PCT (ng/mL) | NA | 9.47 (2.73-14.50) | 25.93 (8.58-82.32) ^†^ | 0.009 |
| BNP (pg/mL) | NA | 188 (120-439) | 377 (239-622) ^†^ | 0.032 |
| CK-MB (ng/mL) | NA | 1.65 (1.25-2.30) | 4.50 (3.50-22.70) ^†^ | <0.001 |
| TnI (ng/mL) | NA | 0.115 (0.049-0.300) | 0.790 (0.410-1.520) ^†^ | <0.001 |
| Lactate (mmol/L) | NA | 1.82 ± 0.80 | 4.89 ± 2.44 ^†^ | <0.001 |
| Ang II (pg/mL) | 47.10 ± 15.41 | 69.82 ± 17.51^*^ | 101.40 ± 33.78^*†^ | <0.001 |
| Ang-(1-7) (pg/mL) | 58.9 (31.8-79.2) | 32.6 (25.8-35.1) | 20.9 (19.2-29.4) ^*^ | <0.001 |
| Ang II/Ang-(1-7) | 0.89 ± 0.36 | 2.24 ± 0.92^*^ | 4.35 ± 2.03^*†^ | <0.001 |

HC: healthy controls; SIC: sepsis-induced cardiomyopathy; COPD: chronic obstructive pulmonary disease; SOFA: Sequential Organ Failure Assessment; APACHE: Acute Physiology: Age and Chronic Health Evaluation; PaO_2_: partial pressure of oxygen in arterial blood; FiO_2_, fraction of inspired oxygen; MAP: mean arterial pressure; TBIL: total bilirubin; WBC: white blood cell count; AST: aspartate aminotransferase; CRP: C-reaction protein; PCT: procalcitonin; BNP: B-type natriuretic peptide; CK-MB: creatine kinase-MB; TnI: Troponin I; Ang II: angiotensin II; Ang-(1-7): angiotensin-(1-7). ^*^*p* < 0.05 vs. HC group; ^†^*p* < 0.05 vs. non-SIC group.

;
