# Supplementary material for: Angiotensin-(1–7) ameliorates sepsis-induced cardiomyopathy by alleviating inflammatory response and mitochondrial damage through the NF-κB and MAPK pathways
Source: J Transl Med. 2023 Jan 2;21:2. doi: 10.1186/s12967-022-03842-5 (PMC9807106; doi:10.1186/s12967-022-03842-5)
Supplement: Supplementary file 3 — Additional file 3: Fig S1. Ang-(1–7) and Ang II/Ang-(1–7) levels in the serum of each group of mice. Fig S2. Effect of Ang-(1–7) on LPS-mediated NF-κB nuclear translocation. Fig S3. Ang-(1–7) alleviates H9c2 cells apoptosis induced by LPS-mediated inflammatory response through the NF-κB and MAPK signaling pathways. [file 12967_2022_3842_MOESM3_ESM.docx]

**
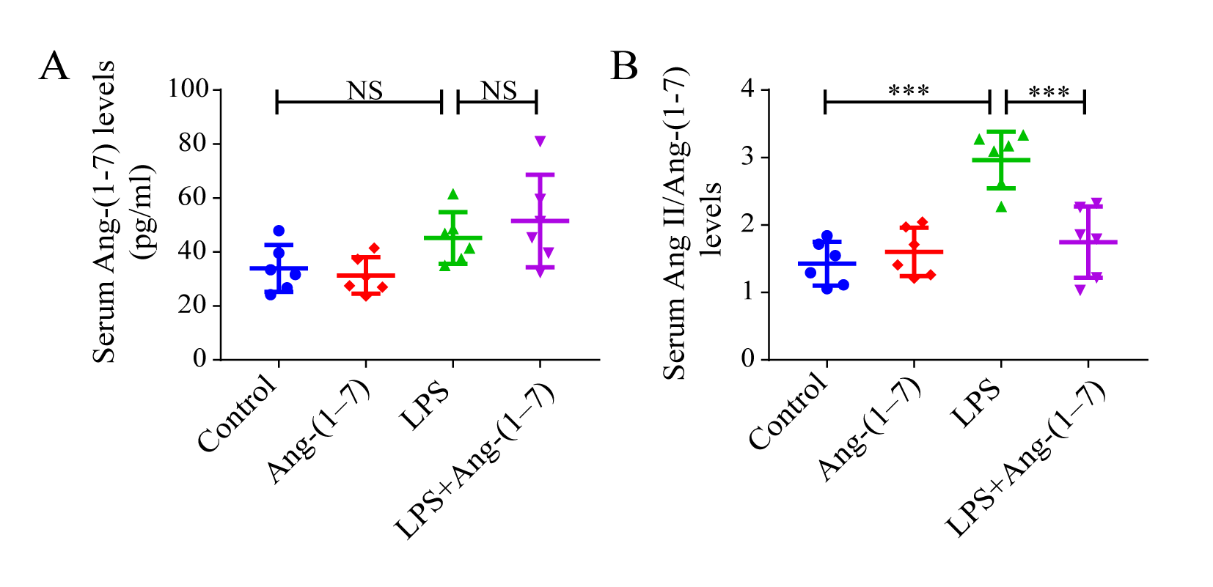
**

**Fig S1. Ang-(1-7) and Ang II/Ang-(1-7) levels in the serum of each group of mice**

(**A-B**) The levels of Ang-(1-7) and Ang II/Ang-(1-7) in the serum of each group of mice were measured by ELISA (n=6). NS: no significant difference, ^***^*p* < 0.001.


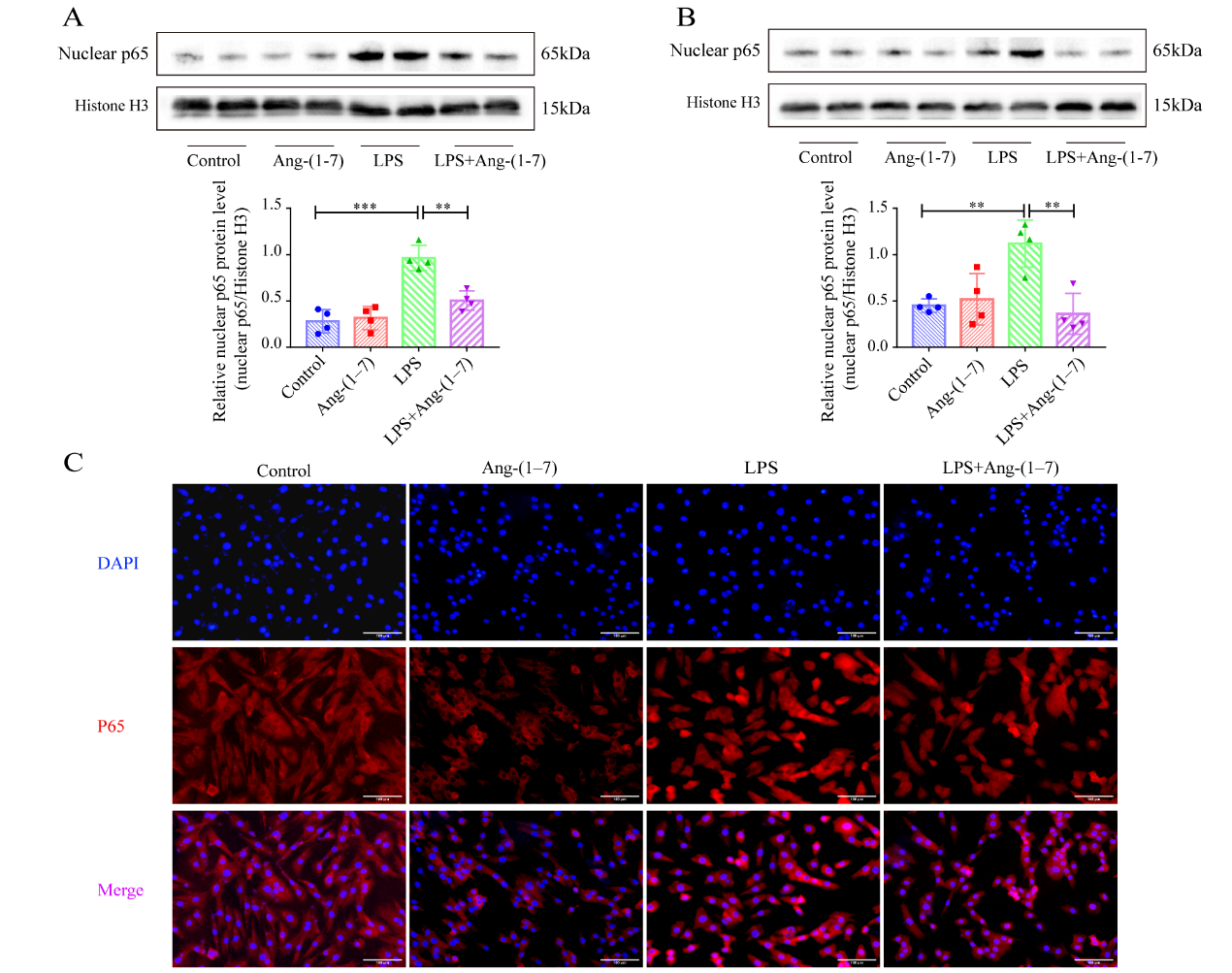


**Fig S2. Effect of Ang-(1-7) on LPS-mediated NF-κB nuclear translocation.**

(**A**) Representative Western blot and quantification of nuclear NF-κB to Histone H3 in myocardial specimens (n=4). (**B**) Representative Western blot and quantification of nuclear NF-κB to Histone H3 in H9c2 cells (n=4). (**C**) Representative image of nuclear translocation of NF-κB p65 in H9c2 cells under fluorescence microscope. (×200, scale bar: 100 μm). ^***^*p* < 0.001, ^**^*p* < 0.01, ^*^*p* < 0.05.


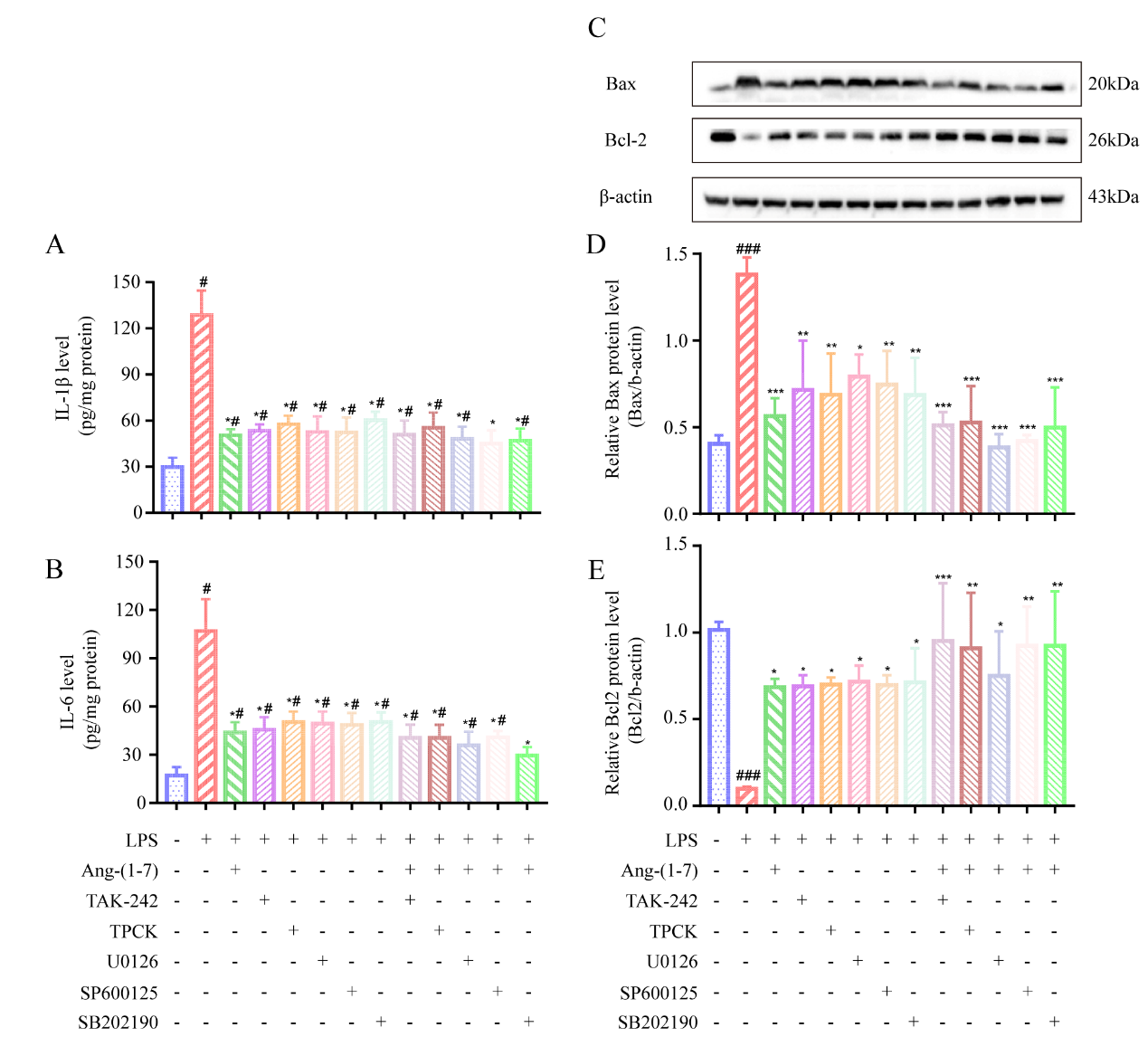


**Fig S3.** **Ang-(1-7) alleviates H9c2 cells apoptosis induced by LPS-mediated inflammatory response through the NF-****κB and MAPK signaling pathways.**

(**A-B**) The concentrations of IL-1β and IL-6 proteins in H9c2 cells of each group were detected by ELISA (n=6). (**C-E**) Representative Western blot and quantification of Bax and Bcl-2 in H9c2 cells of each group (n=3). (**A-E**) TLR4 inhibitor TAK-242 (20 µM), NF-κB inhibitor TPCK (20 µM), ERK inhibitor U0126 (20 µM), JNK inhibitor SP600125 (20 µM), and p38 inhibitor SB202190 (20 µM) were administered to H9c2 cells separately with or without Ang-(1-7) (10^-6^ mol/L) for 1 h before LPS (1 μg/mL) stimulation for 12 h. (**A-B**) ^*^*p* < 0.05 compared with the control group, ^#^*p* < 0.05 compared with the LPS group. (**C-E**) ^###^*p* < 0.001 compared with the control group; ^*^*p* < 0.05, ^**^*p* < 0.01, ^***^*p* < 0.001 compared with the LPS group.
